# Supplementary material for: Ganoderic Acid A and Its Amide Derivatives as Potential Anti-Cancer Agents by Regulating the p53-MDM2 Pathway: Synthesis and Biological Evaluation
Source: Molecules. 2023 Mar 4;28(5):2374. doi: 10.3390/molecules28052374 (PMC10004777; doi:10.3390/molecules28052374)
Supplement: Supplementary file 1 [file molecules-28-02374-s001.zip › molecules-2238873-supplementary.pdf]

Supplementary Materials

Ganoderic Acid A and its amide Derivatives as Potential Anti-cancer Agents by Regulating p53-MDM2 Pathway: Synthesis and Biological Evaluation

Supplementary Figure. S1 The chemical structure of compound A1. Chemical formula $C_{34}H_{53}NO_6$; molecular weight: 571.80 4

Supplementary Figure. S2 The ^{1}H NMR spectra of compound A1 4

Supplementary Figure. S3 The ^{13}C NMR spectra of compound A1 5

Supplementary Figure. S4 The HRMS spectra of compound A1 5

Supplementary Figure. S5 The chemical structure of compound A2. Chemical formula $C_{36}H_{57}NO_6$; molecular weight 599.85 6

Supplementary Figure. S6 The ^{1}H NMR spectra of compound A2 6

Supplementary Figure. S7 The ^{13}C NMR spectra of compound A2 7

Supplementary Figure. S8 The HRMS spectra of compound A2 7

Supplementary Figure. S9 The chemical structure of compound A3. Chemical formula $C_{37}H_{51}NO_6$; molecular weight: 605.82 8

Supplementary Figure. S10 The ^{1}H NMR spectra of compound A3 8

Supplementary Figure. S11 The ^{13}C NMR spectra of compound A3 9

Supplementary Figure. S12 The HRMS spectra of compound A3 9

Supplementary Figure. S13 The chemical structure of compound A4. Chemical formula $C_{36}H_{48}ClNO_6$; molecular weight: 626.23 10

Supplementary Figure. S14 The ^{1}H NMR spectra of compound A4 10

Supplementary Figure. S15 The ^{13}C NMR spectra of compound A4 11

Supplementary Figure. S16 The ^{1}H NMR spectra of compound A4 11

Supplementary Figure. S17 The chemical structure of compound A5. Chemical formula $C_{38}H_{53}NO_6$; molecular weight: 619.84 12

Supplementary Figure. S18 The ^{1}H NMR spectra of compound A5 12

Supplementary Figure. S19 The ^{13}C NMR spectra of compound A5 13

Supplementary Figure. S20 The HRMS spectra of compound A5 13

Supplementary Figure. S21 The chemical structure of compound A6. Chemical formula $C_{37}H_{50}FNO_6$; molecular weight: 623.81 14

Supplementary Figure. S22 The ^{1}H NMR spectra of compound A6 14

Supplementary Figure. S23 The ^{13}C NMR spectra of compound A6 15

Supplementary Figure. S24 The HRMS spectra of compound A6 15

Supplementary Figure. S25 The chemical structure of compound A7. Chemical formula $C_{37}H_{50}ClNO_6$; molecular weight: 640.26 16

Supplementary Figure. S26 The ^{1}H NMR spectra of compound A7 16

Supplementary Figure. S27 The ^{13}C NMR spectra of compound A7 17

Supplementary Figure. S28 The HRMS spectra of compound A7 17

Supplementary Figure. S29 The chemical structure of compound A8. Chemical formula $C_{37}H_{49}Cl_2NO_6$; molecular weight: 674.70 18

Supplementary Figure. S30 The ^{1}H NMR spectra of compound A8 18

Supplementary Figure. S31 The ^{13}C NMR spectra of compound A8 19

Supplementary Figure. S32 The HRMS spectra of compound A8 19

Supplementary Figure. S33 The chemical structure of compound A9. Chemical formula $C_{39}H_{53}NO_6$; molecular weight: 631.85 20

Supplementary Figure. S34 The ^{1}H NMR spectra of compound A9 20

Supplementary Figure. S35 The ^{13}C NMR spectra of compound A9 21

Supplementary Figure. S36 The HRMS spectra of compound A9 21

Supplementary Figure. S37 The chemical structure of compound A10. Chemical formula $C_{39}H_{55}NO_6$; molecular weight: 633.87 22

Supplementary Figure. S38 The ^{1}H NMR spectra of compound A10 22

Supplementary Figure. S39 The ^{13}C NMR spectra of compound A10 23

Supplementary Figure. S40 The HRMS spectra of compound A10 23

Supplementary Figure. S41 The chemical structure of compound A11. Chemical formula $C_{38}H_{52}ClNO_6$; molecular weight: 637.83 24

Supplementary Figure. S42 The ^{1}H NMR spectra of compound A11 24

Supplementary Figure. S43 The ^{13}C NMR spectra of compound A11 25

Supplementary Figure. S44 The HRMS spectra of compound A11 25

Supplementary Figure. S45 The chemical structure of compound A12. Chemical formula $C_{38}H_{52}ClNO_6$; molecular weight: 654.29 26

Supplementary Figure. S46 The ^{1}H NMR spectra of compound A12 26

Supplementary Figure. S47 The ^{13}C NMR spectra of compound A12 27

Supplementary Figure. S48 The HRMS spectra of compound A12 27

Supplementary Figure. S49 The chemical structure of compound A13. Chemical formula $C_{35}H_{54}N_2O_6$; molecular weight: 598.83 28

Supplementary Figure. S50 The ^{1}H NMR spectra of compound A13 28

Supplementary Figure. S51 The ^{13}C NMR spectra of compound A13 29

Supplementary Figure. S52 The HRMS spectra of compound A13 29

Supplementary Figure. S53 The chemical structure of compound A14. Chemical formula $C_{36}H_{56}N_2O_6$; molecular weight: 612.85 30

Supplementary Figure. S54 The ^{1}H NMR spectra of compound A14 30

Supplementary Figure. S55 The ^{13}C NMR spectra of compound A14 31

Supplementary Figure. S56 The HRMS spectra of compound A14 31

Supplementary Figure. S57 The chemical structure of compound A15. Chemical formula $C_{40}H_{56}N_2O$; molecular weight: 660.90 32

Supplementary Figure. S58 The ^{1}H NMR spectra of compound A15 32

Supplementary Figure. S59 The ^{13}C NMR spectra of compound A15 33

Supplementary Figure. S60 The HRMS spectra of compound A15 33

The NMR and HRMS spectra of all the new synthesized compounds.

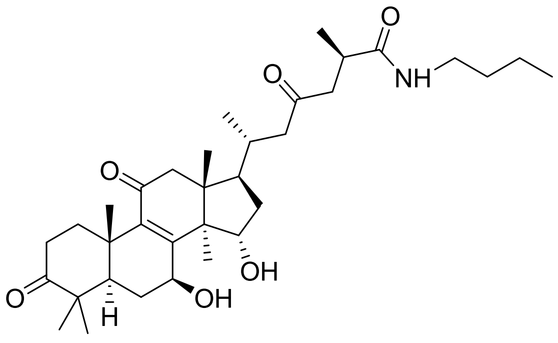

Supplementary Figure. S1 The chemical structure of compound **A1.** Chemical formula $C_{34}H_{53}NO_6$; molecular weight: 571.80

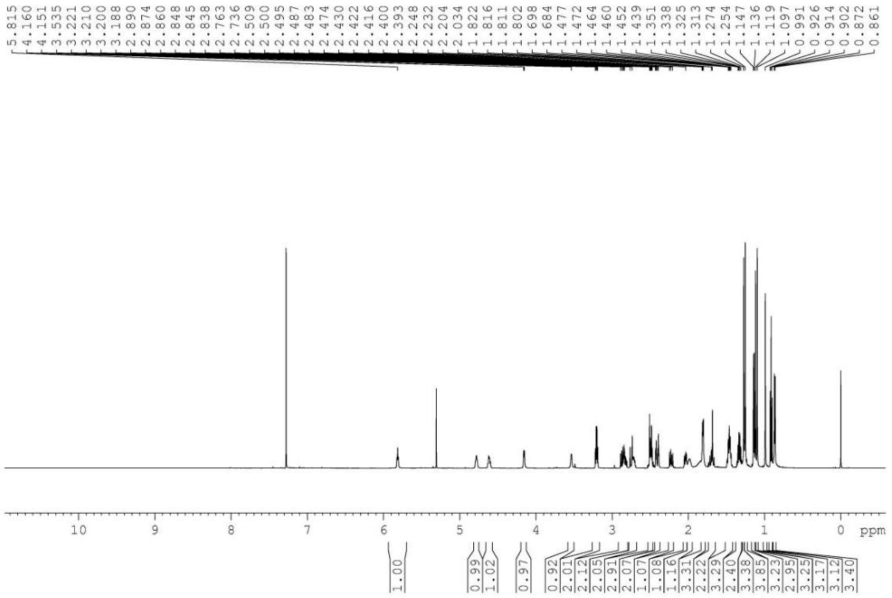

Supplementary Figure. S2 The ^{1}H NMR spectra of compound **A1**

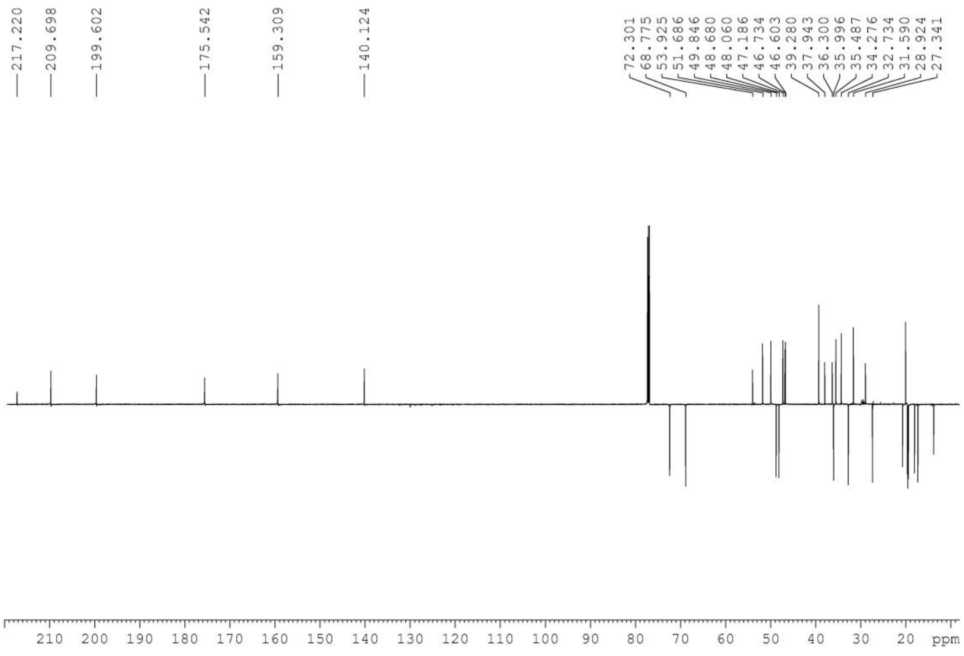

Supplementary Figure. S3 The ^{13}C NMR spectra of compound **A1**

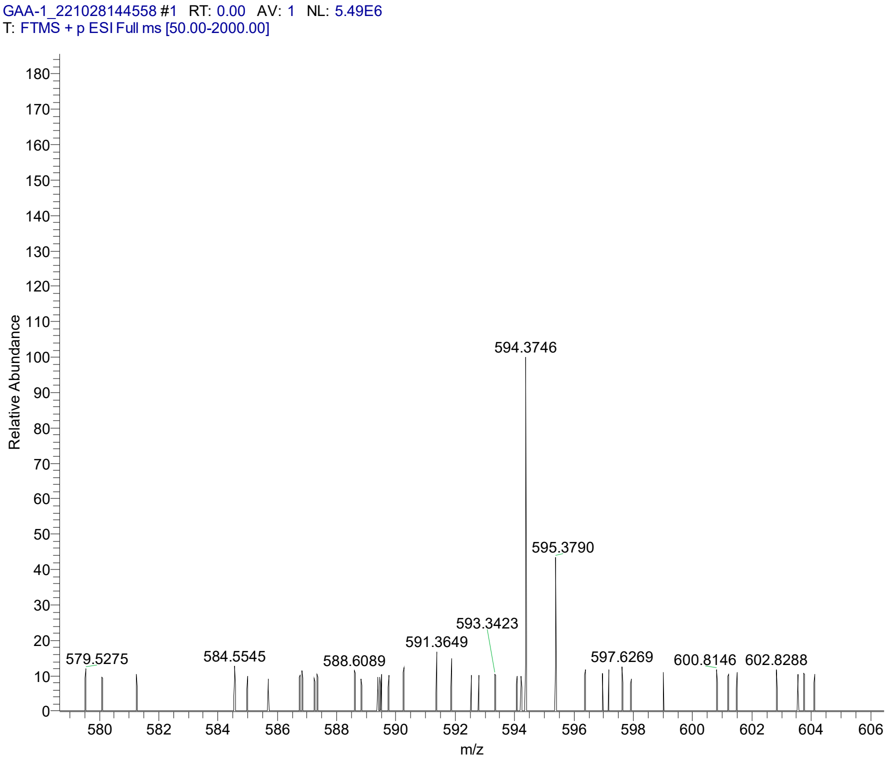

Supplementary Figure. S4 The HRMS spectra of compound **A1**

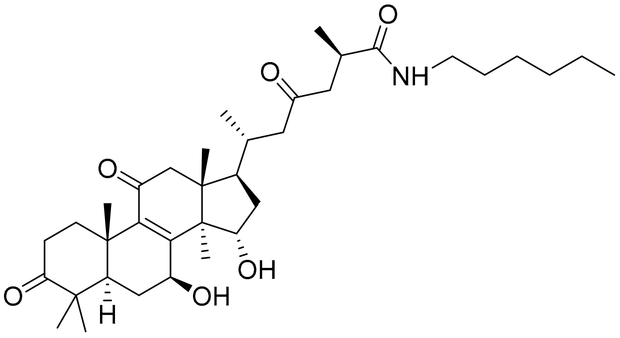

Supplementary Figure. S5 The chemical structure of compound **A2.** Chemical formula $C_{36}H_{57}NO_6$; molecular weight 599.85

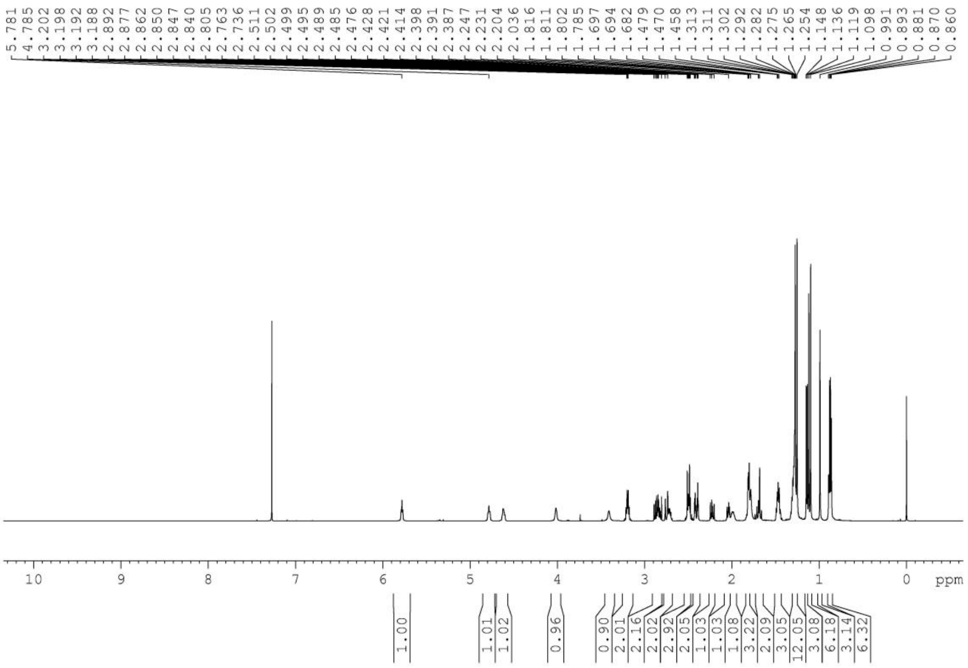

Supplementary Figure. S6 The ^{1}H NMR spectra of compound **A2**

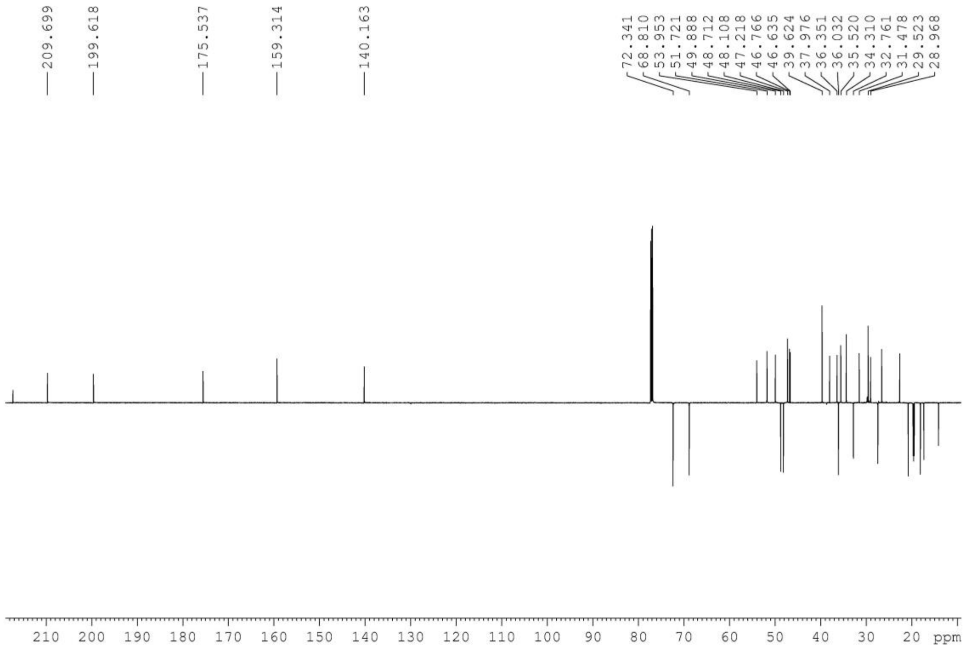

Supplementary Figure. S7 The ^{13}C NMR spectra of compound **A2**

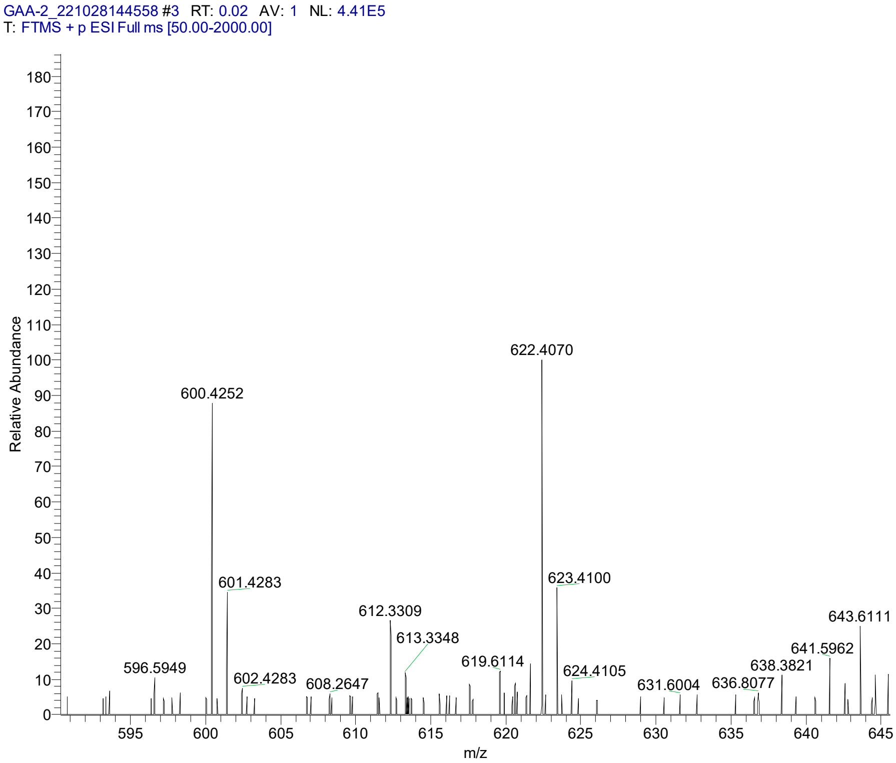

Supplementary Figure. S8 The HRMS spectra of compound **A2**

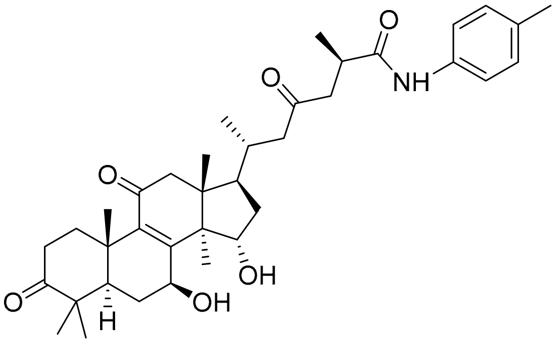

Supplementary Figure. S9 The chemical structure of compound **A3.** Chemical formula $C_{37}H_{51}NO_6$; molecular weight: 605.82

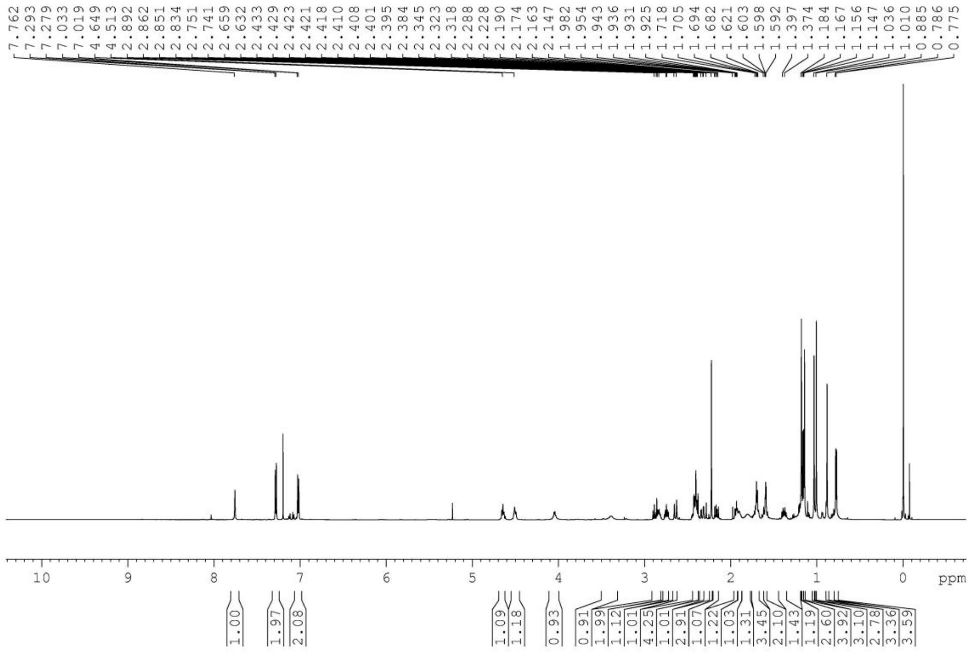

Supplementary Figure. S10 The ^{1}H NMR spectra of compound **A3**

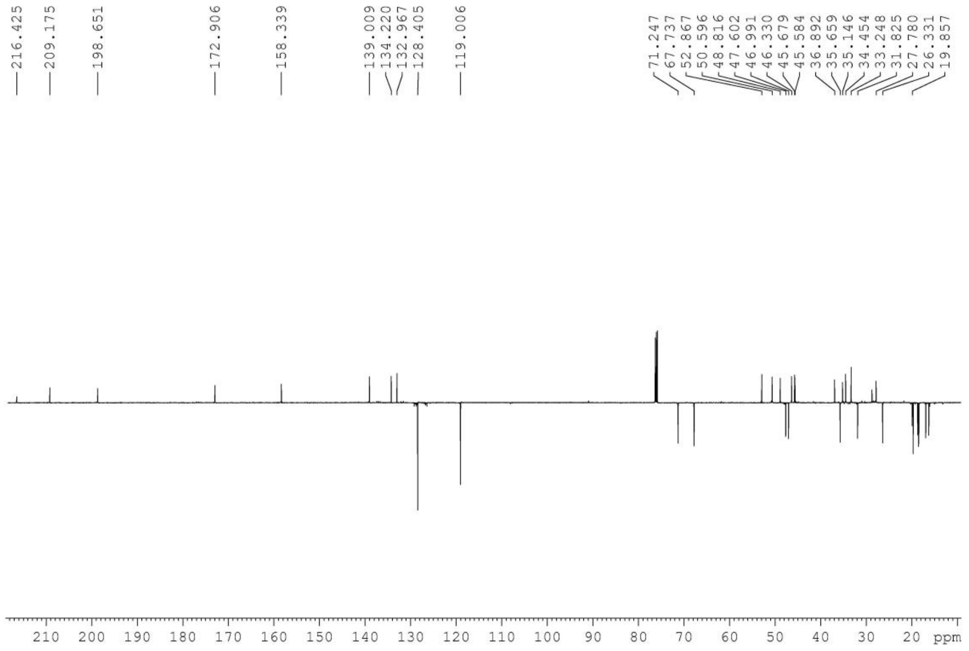

Supplementary Figure. S11 The ^{13}C NMR spectra of compound **A3**

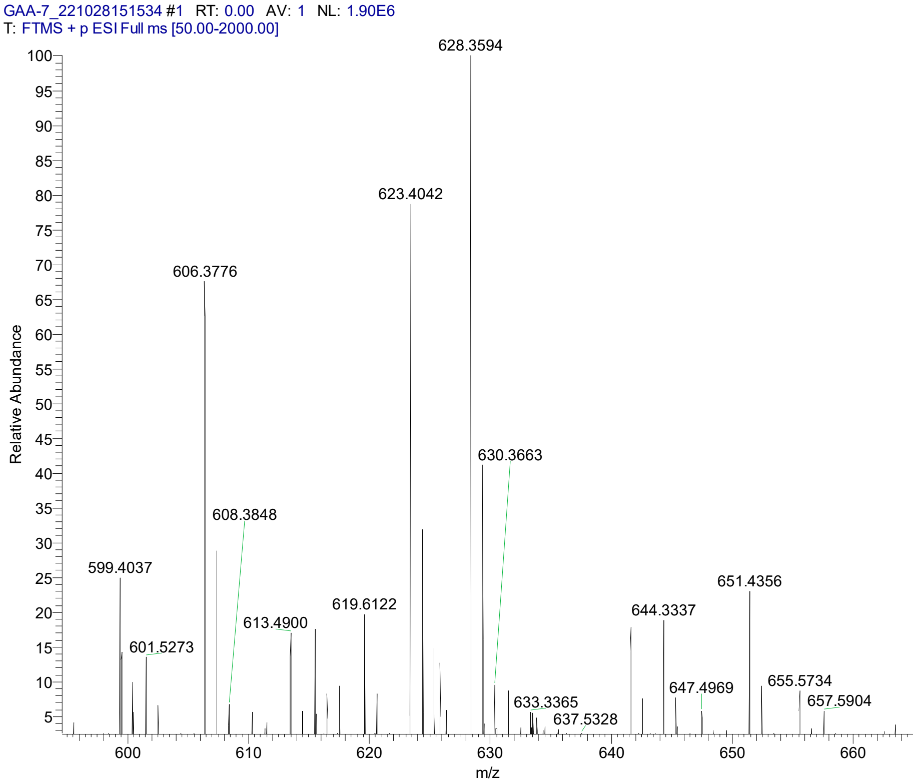

Supplementary Figure. S12 The HRMS spectra of compound **A3**

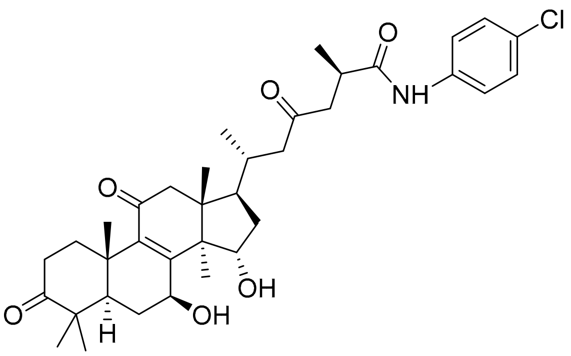

Supplementary Figure. S13 The chemical structure of compound **A4**. Chemical formula $C_{36}H_{48}ClNO_6$; molecular weight: 626.23

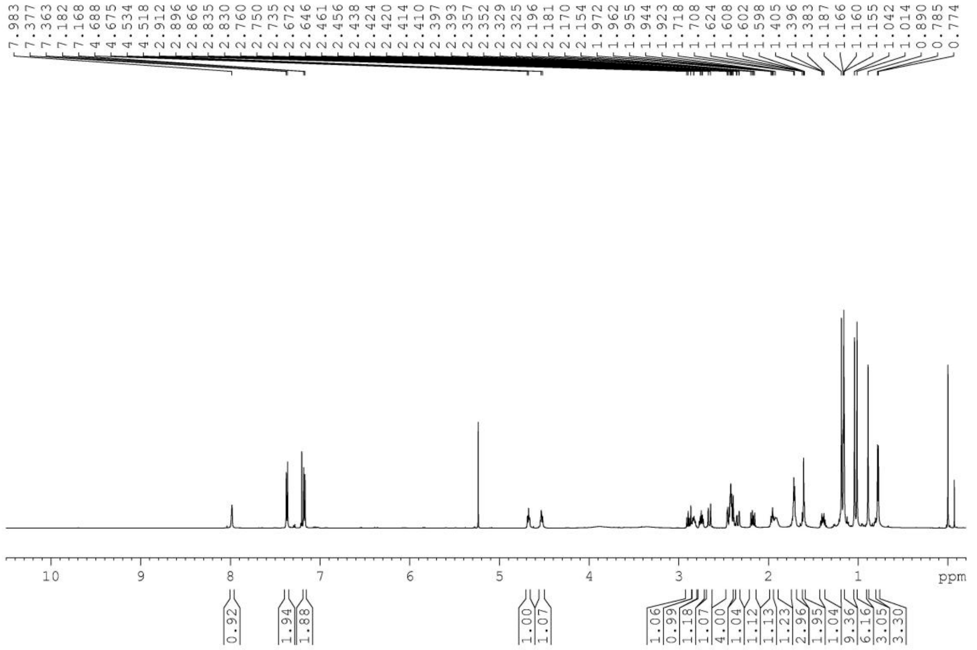

Supplementary Figure. S14 The ^{1}H NMR spectra of compound **A4**

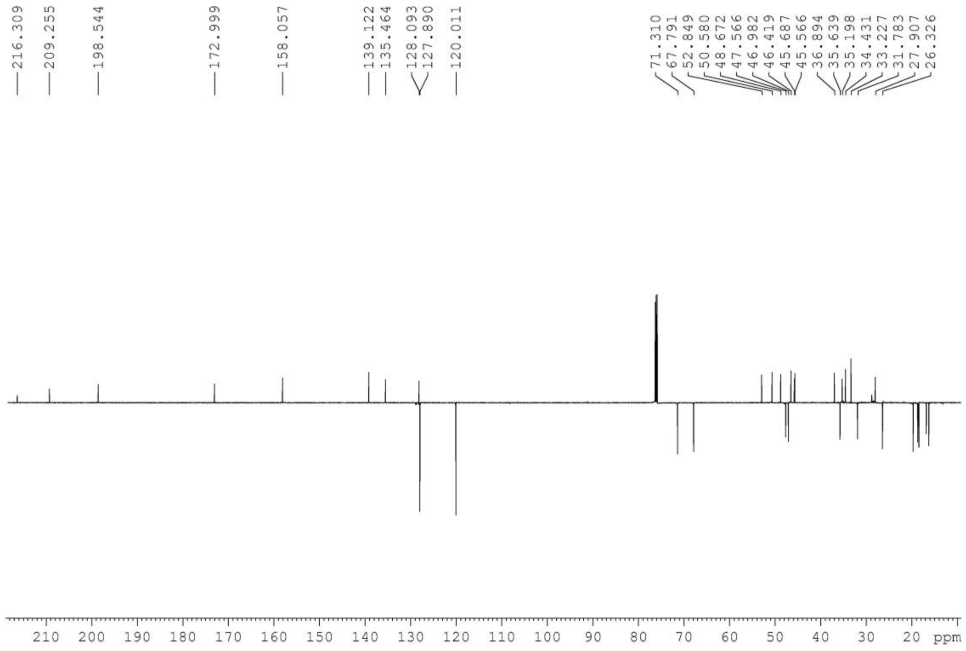

Supplementary Figure. S15 The ^{13}C NMR spectra of compound **A4**

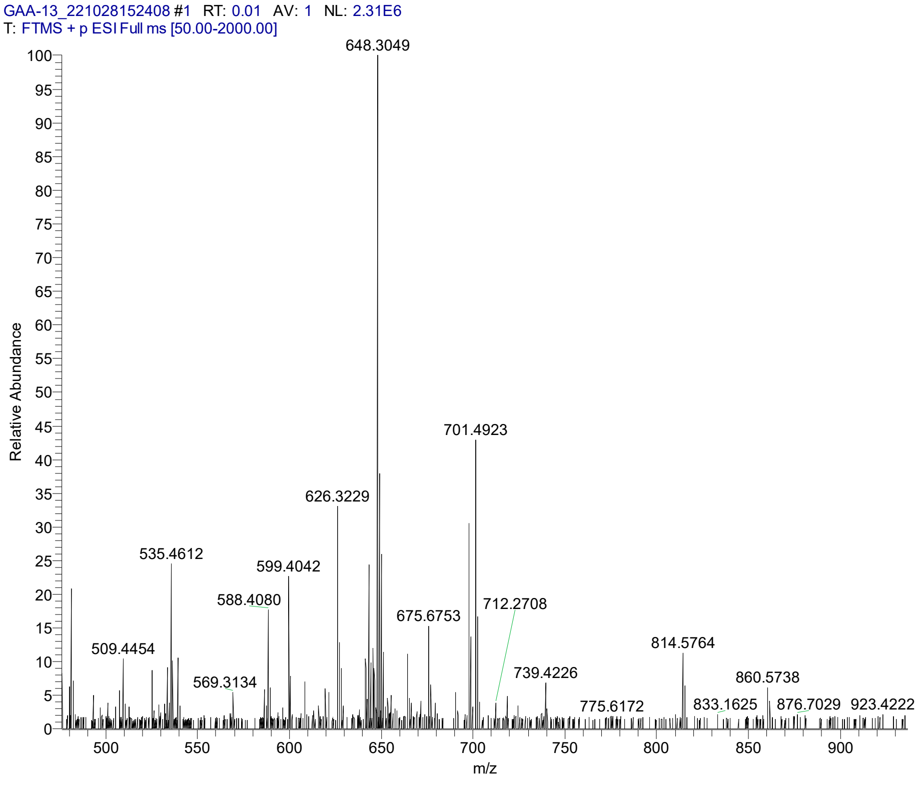

Supplementary Figure. S16 The ^{1}H NMR spectra of compound **A4**

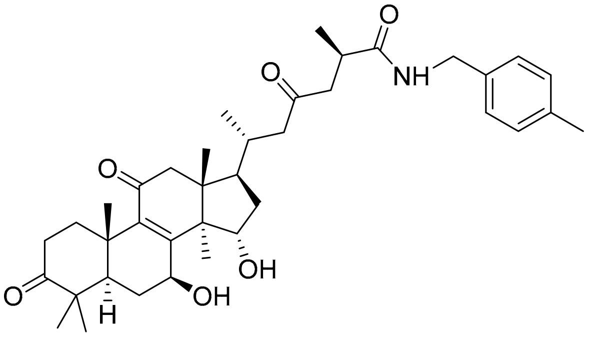

Supplementary Figure. S17 The chemical structure of compound **A5.** Chemical formula $C_{38}H_{53}NO_6$; molecular weight: 619.84

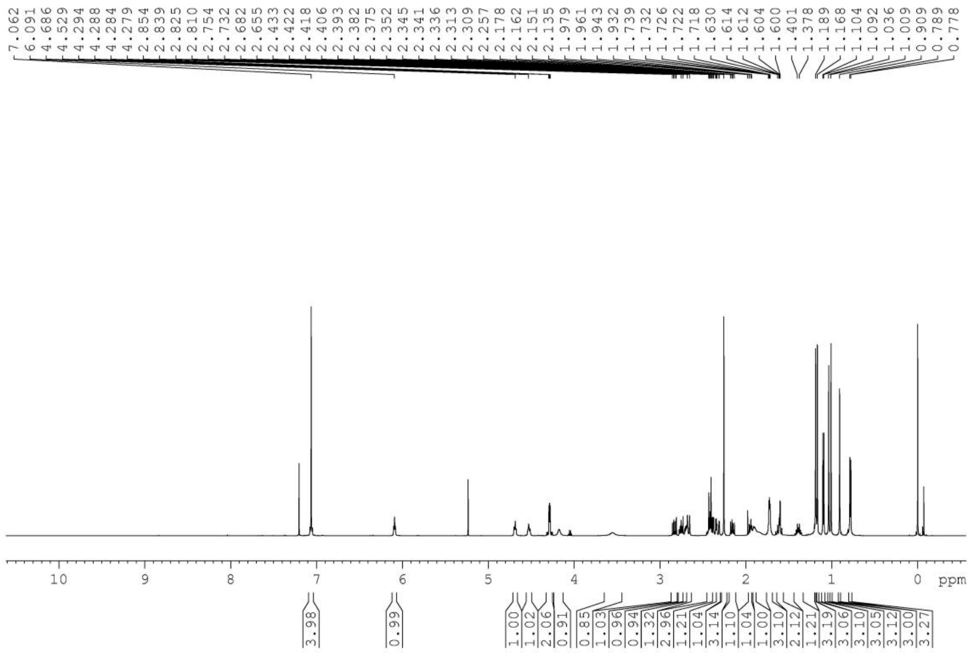

Supplementary Figure. S18 The ^{1}H NMR spectra of compound **A5**

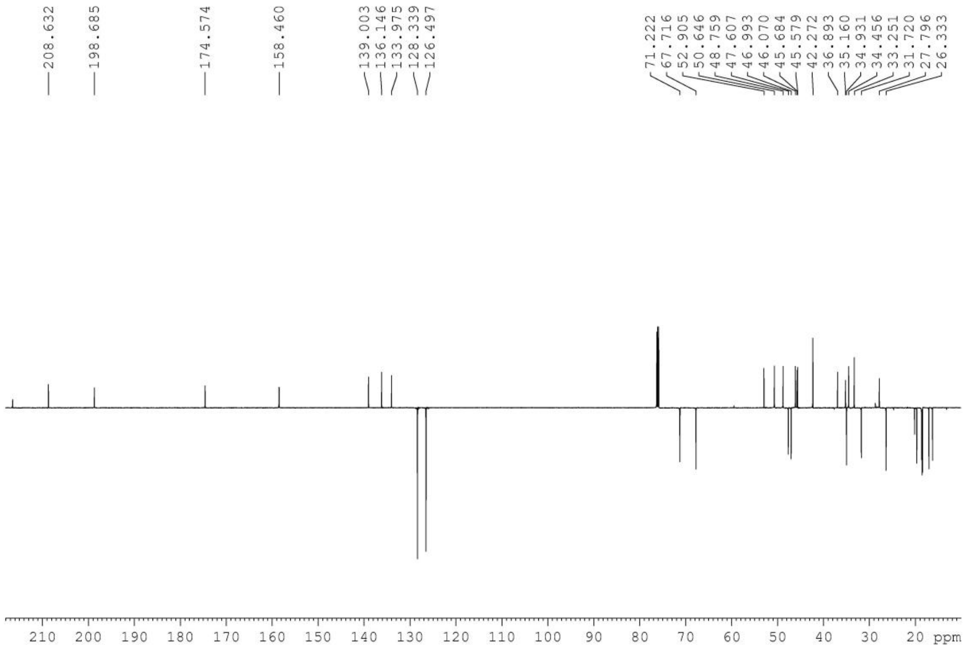

Supplementary Figure. S19 The ^{13}C NMR spectra of compound **A5**

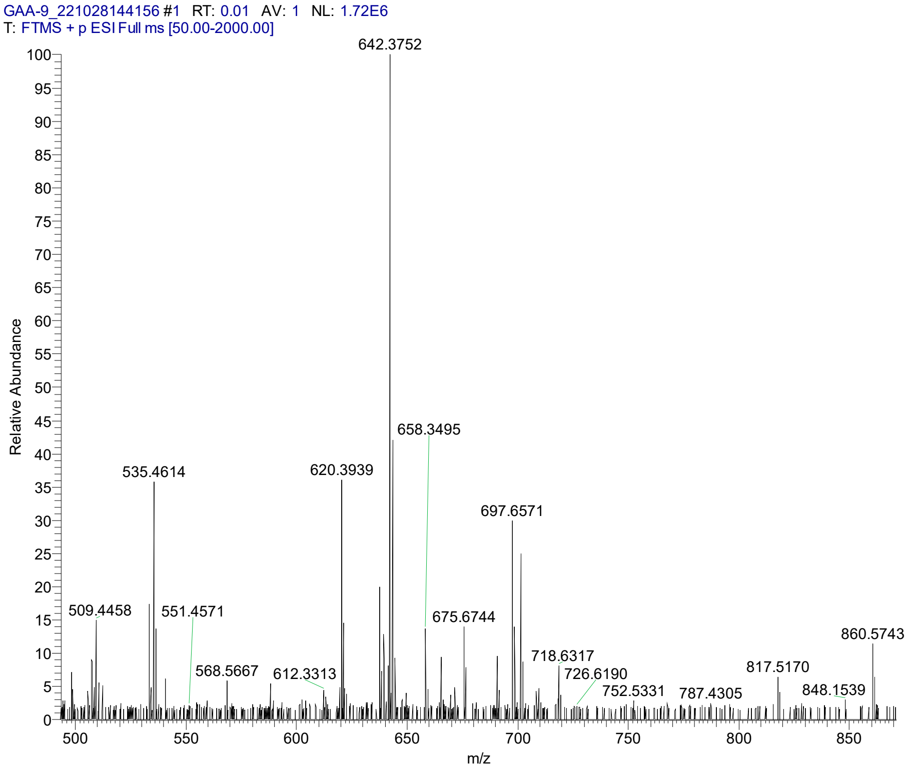

Supplementary Figure. S20 The HRMS spectra of compound **A5**

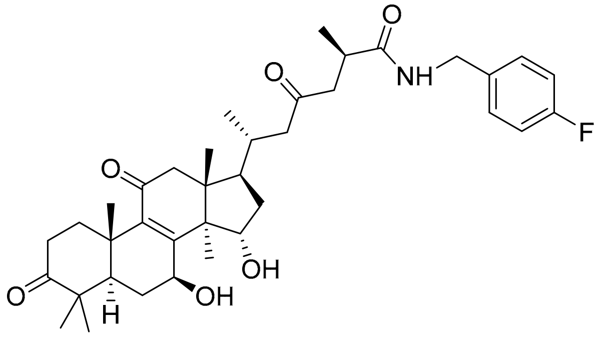

Supplementary Figure. S21 The chemical structure of compound **A6**. Chemical formula $C_{37}H_{50}FNO_6$; molecular weight: 623.81

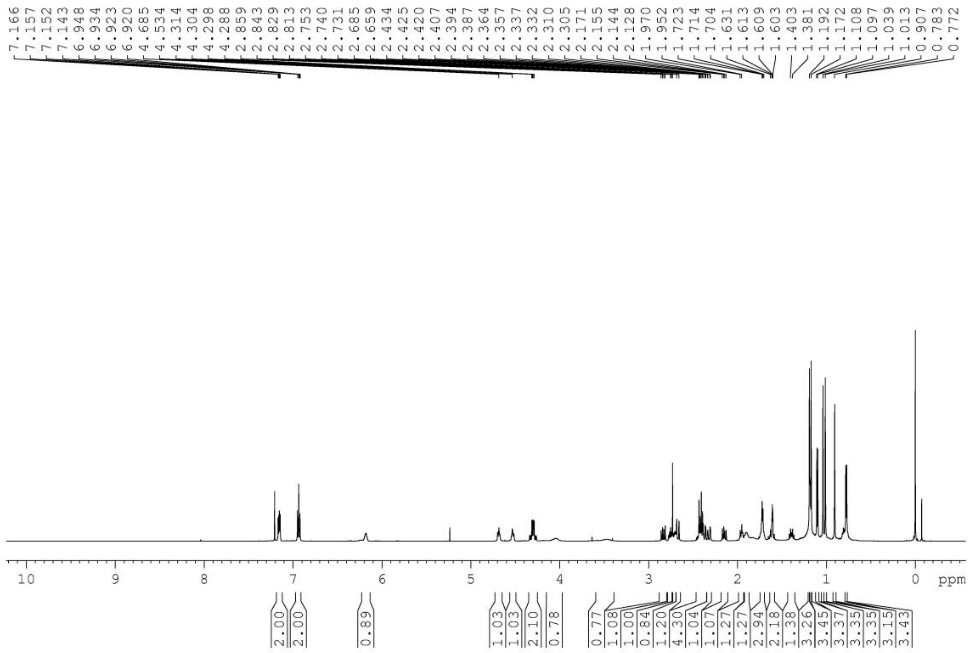

Supplementary Figure. S22 The ^{1}H NMR spectra of compound **A6**

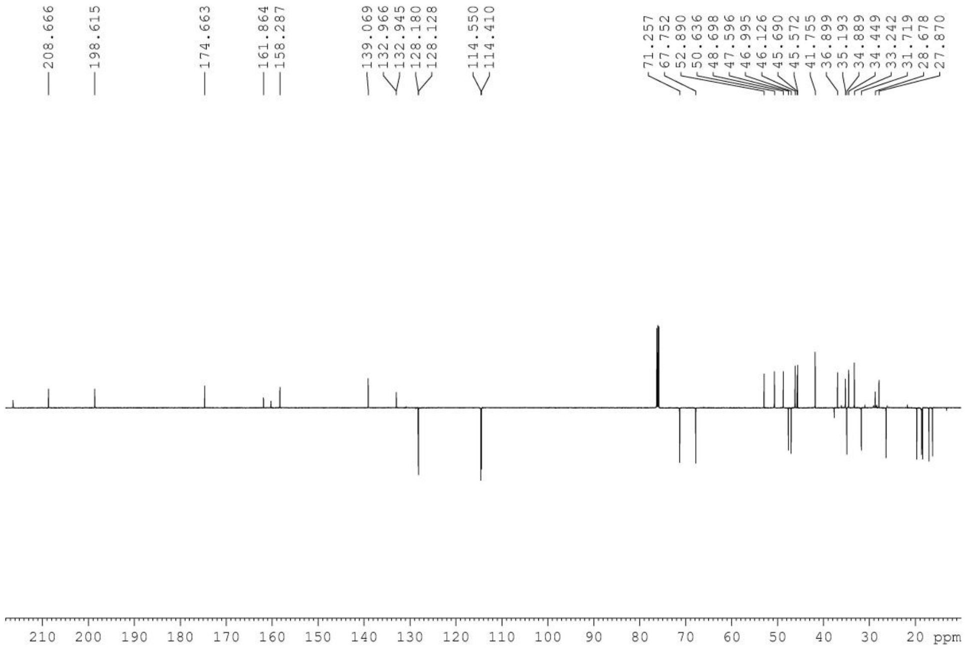

Supplementary Figure. S23 The ^{13}C NMR spectra of compound **A6**

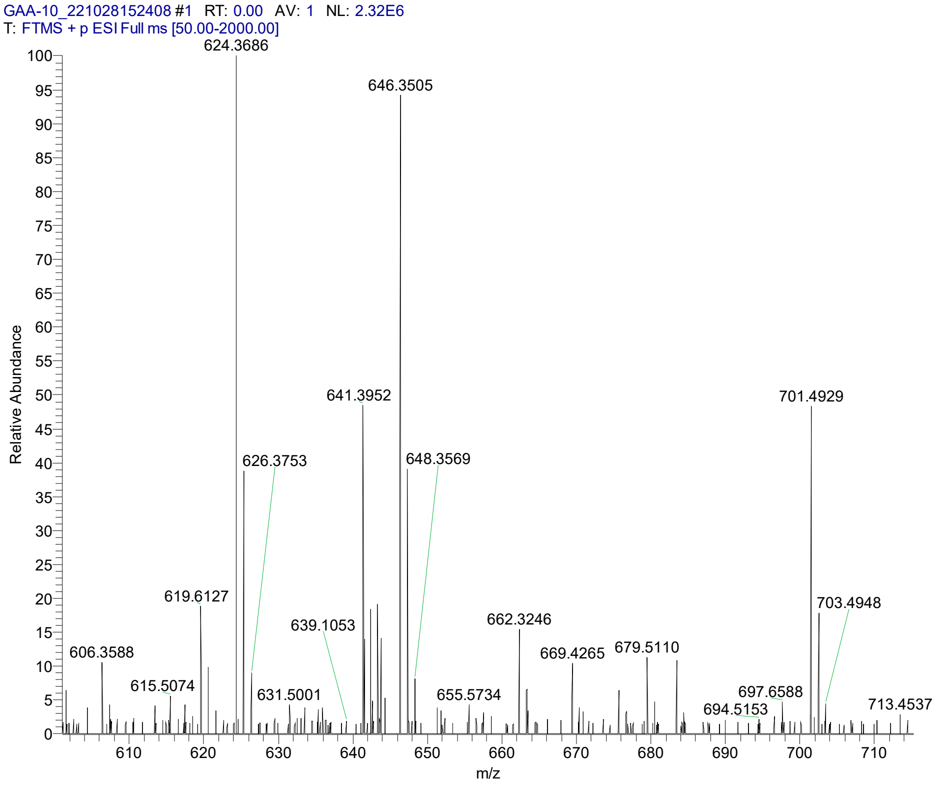

Supplementary Figure. S24 The HRMS spectra of compound **A6**

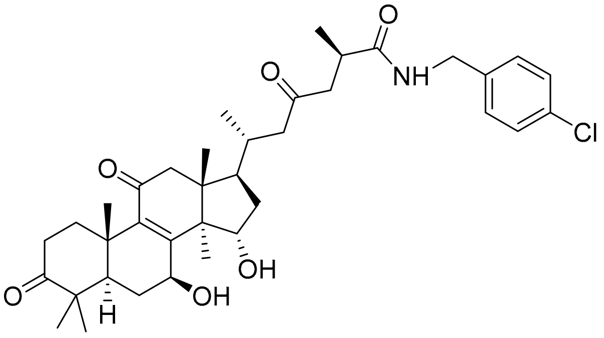

Supplementary Figure. S25 The chemical structure of compound **A7.** Chemical formula $C_{37}H_{50}ClNO_6$; molecular weight: 640.26

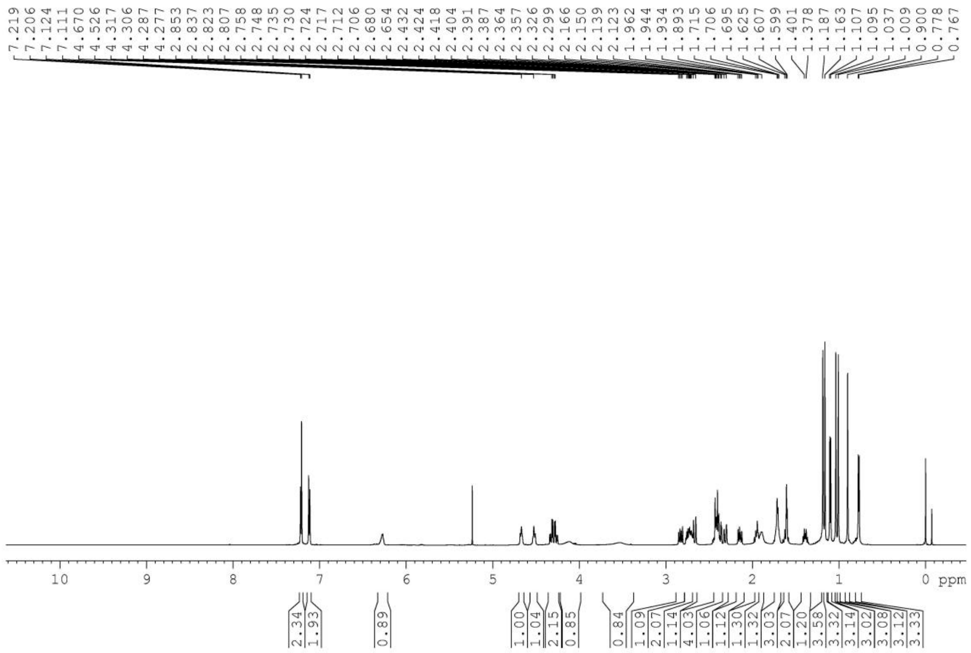

Supplementary Figure. S26 The ^{1}H NMR spectra of compound **A7**

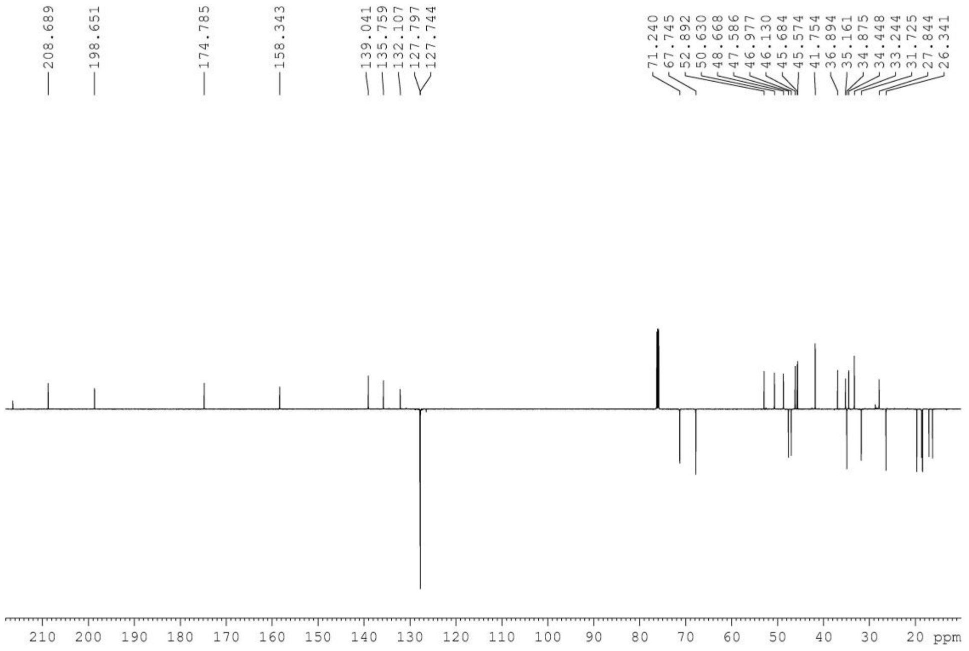

Supplementary Figure. S27 The ^{13}C NMR spectra of compound **A7**

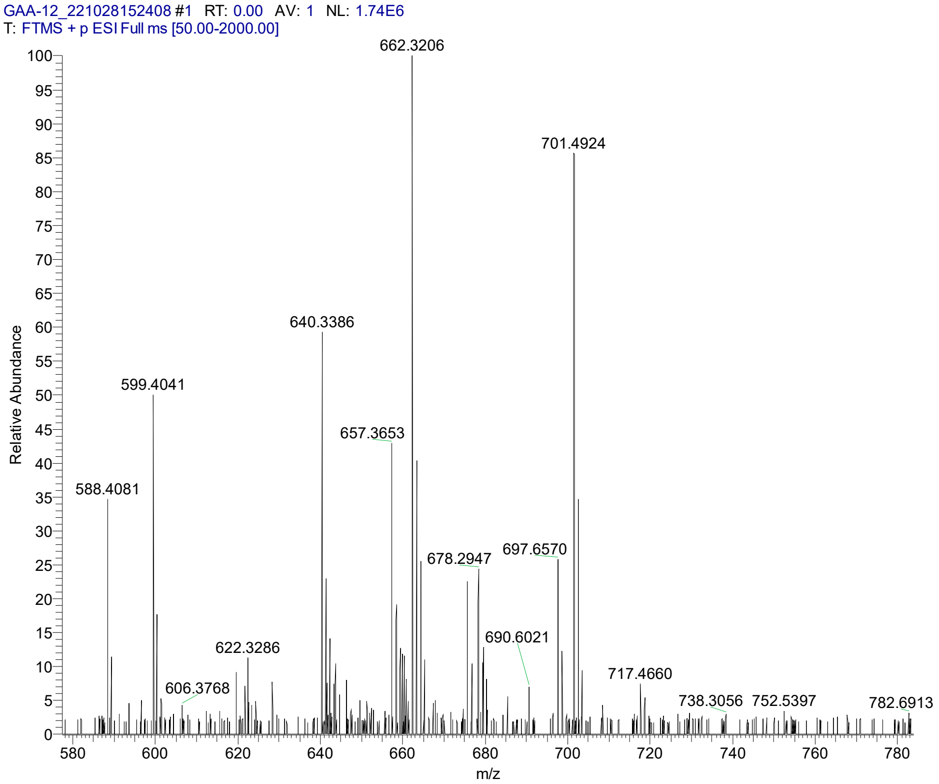

Supplementary Figure. S28 The HRMS spectra of compound **A7**

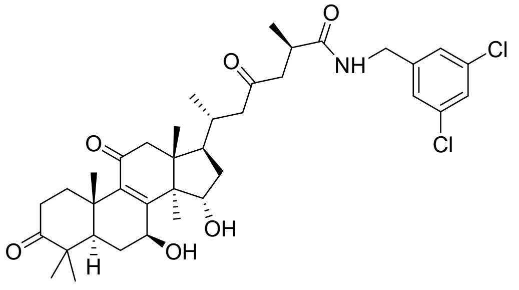

Supplementary Figure. S29 The chemical structure of compound **A8.** Chemical formula $C_{37}H_{49}Cl_2NO_6$; molecular weight: 674.70

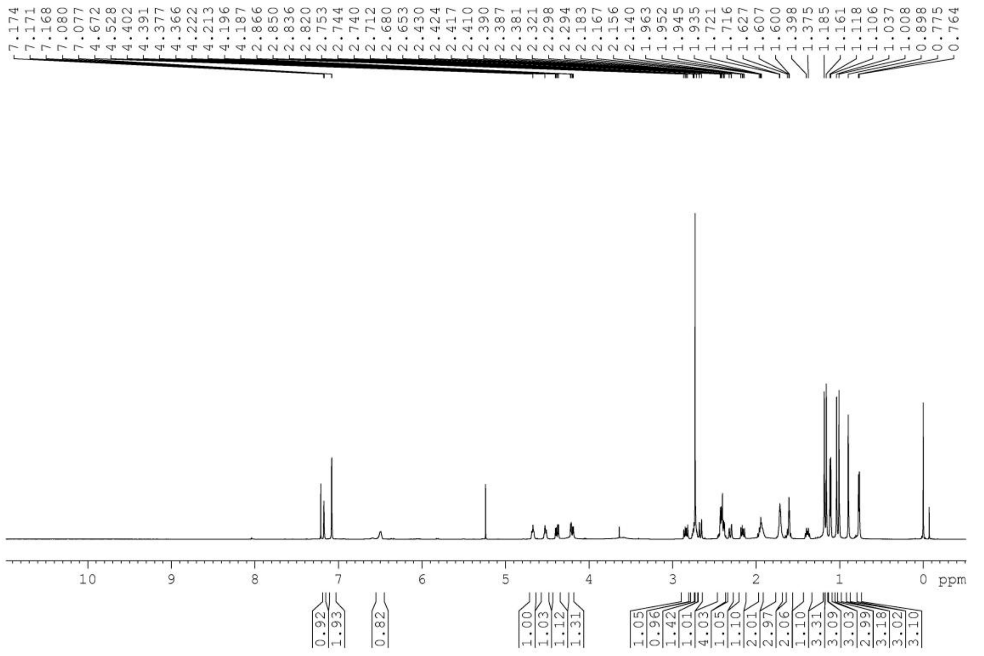

Supplementary Figure. S30 The ^{1}H NMR spectra of compound **A8**

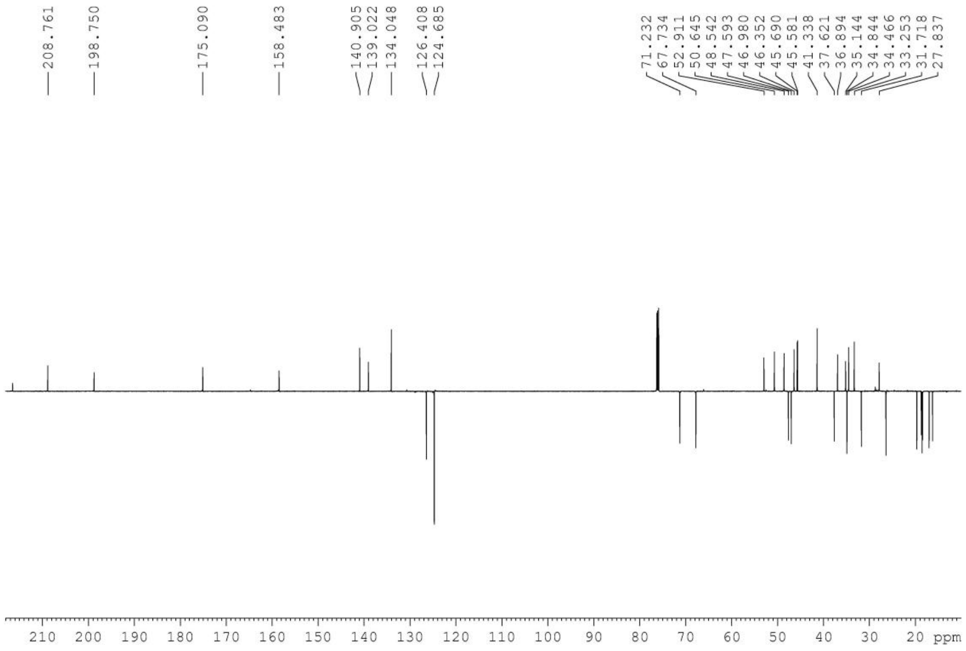

Supplementary Figure. S31 The ^{13}C NMR spectra of compound **A8**

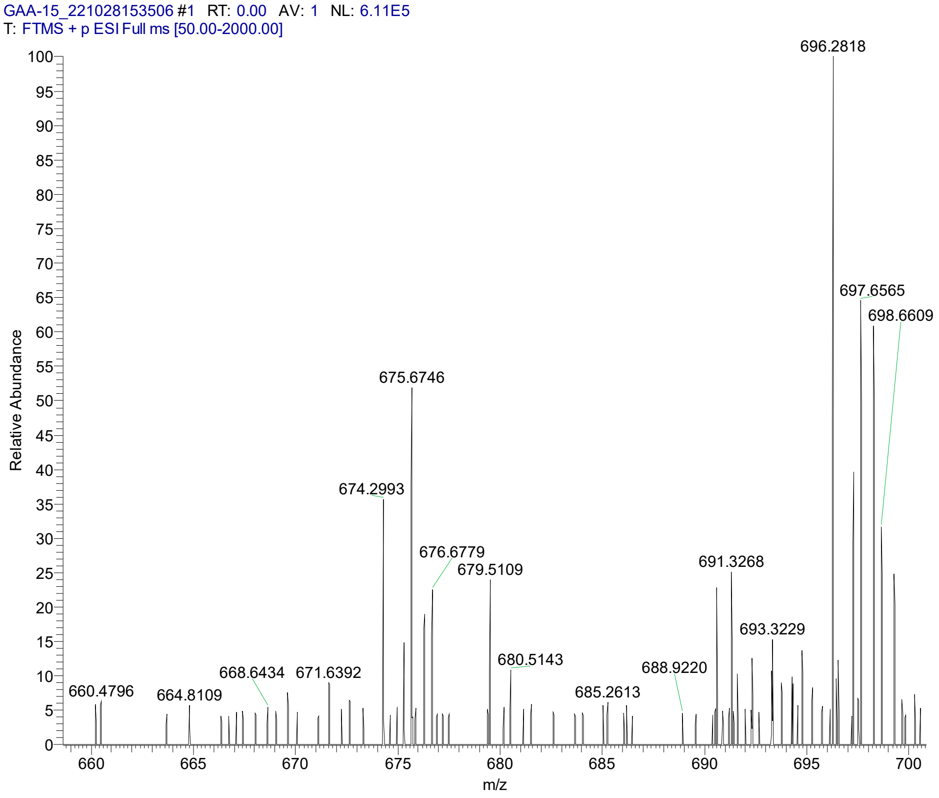

Supplementary Figure. S32 The HRMS spectra of compound **A8**

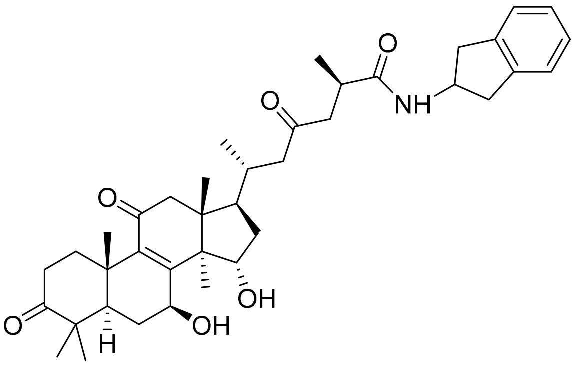

Supplementary Figure. S33 The chemical structure of compound **A9.** Chemical formula $C_{39}H_{53}NO_6$; molecular weight: 631.85

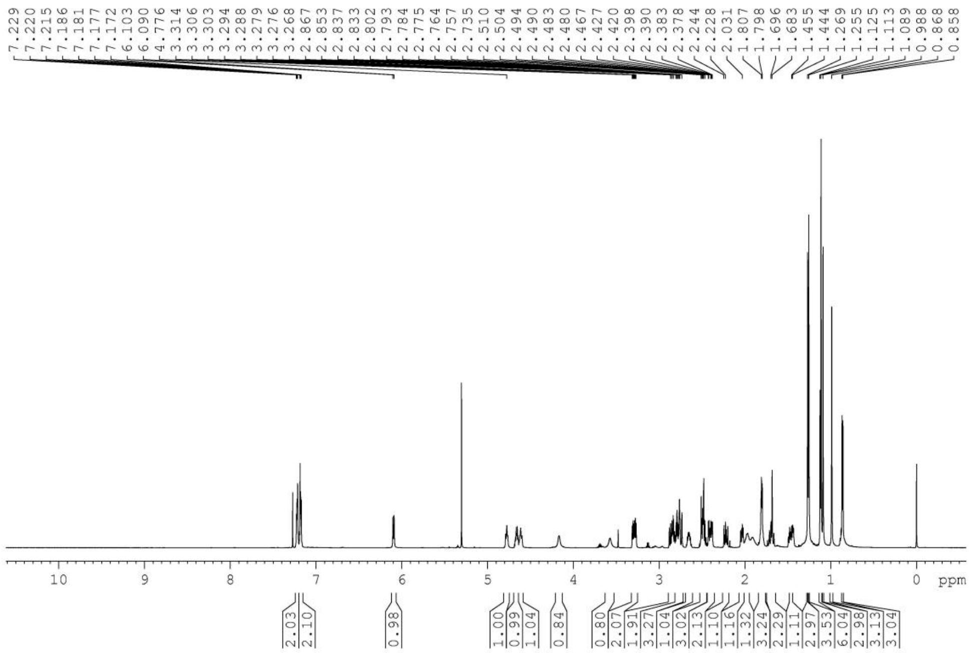

Supplementary Figure. S34 The ^{1}H NMR spectra of compound **A9**

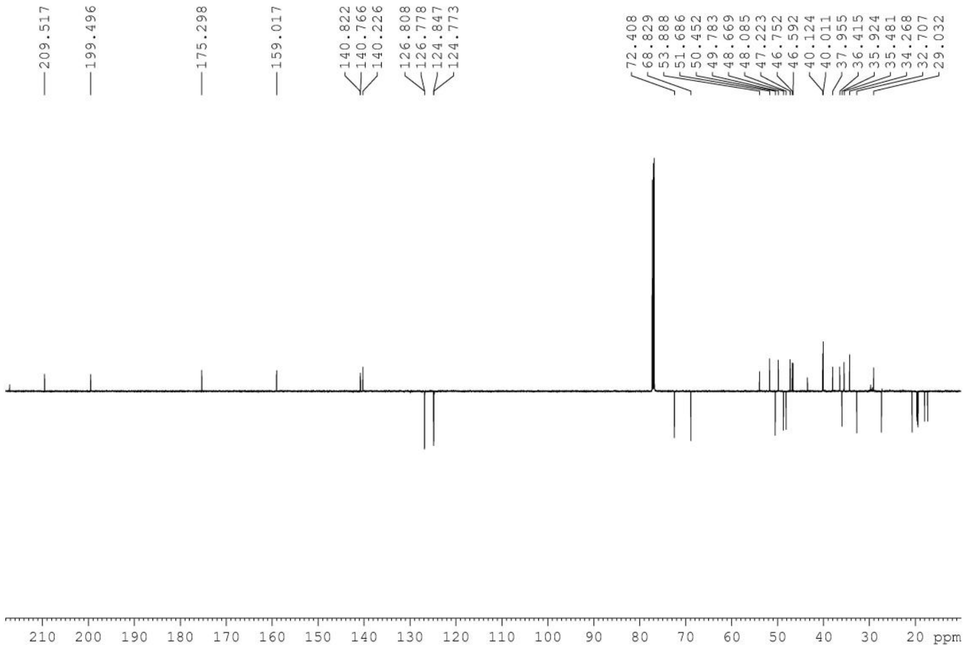

Supplementary Figure. S35 The ^{13}C NMR spectra of compound **A9**

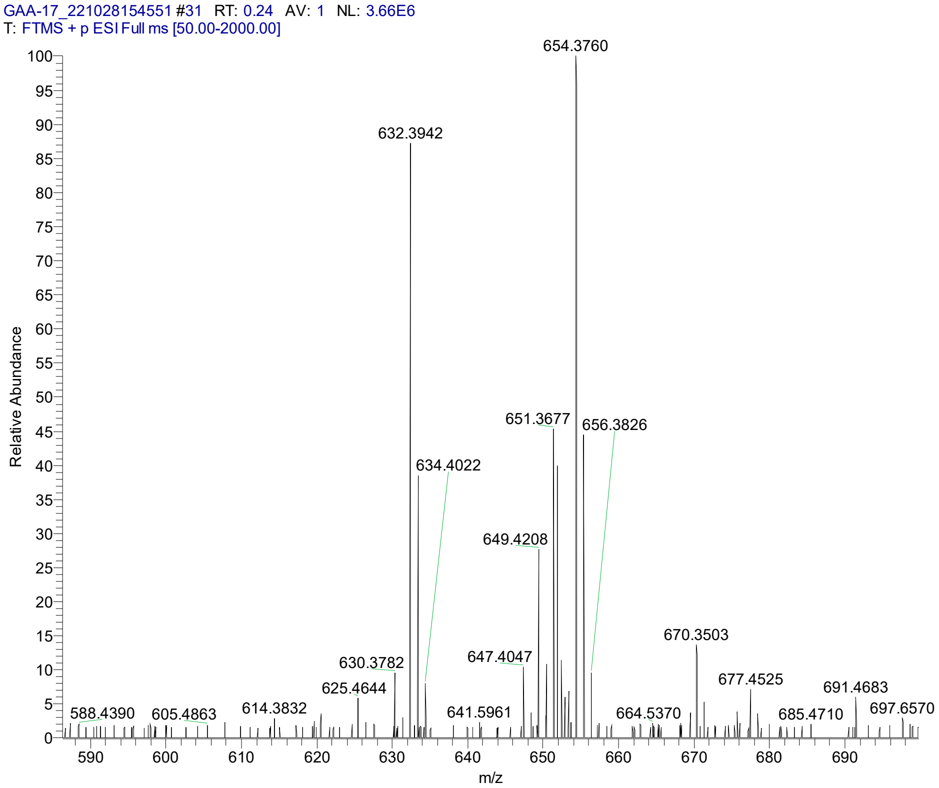

Supplementary Figure. S36 The HRMS spectra of compound **A9**

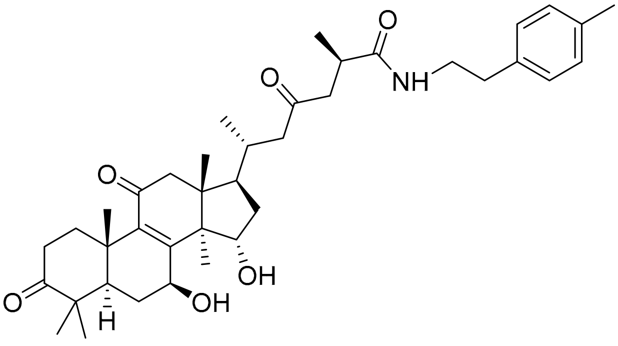

Supplementary Figure. S37 The chemical structure of compound **A10**. Chemical formula $C_{39}H_{55}NO_6$; molecular weight: 633.87

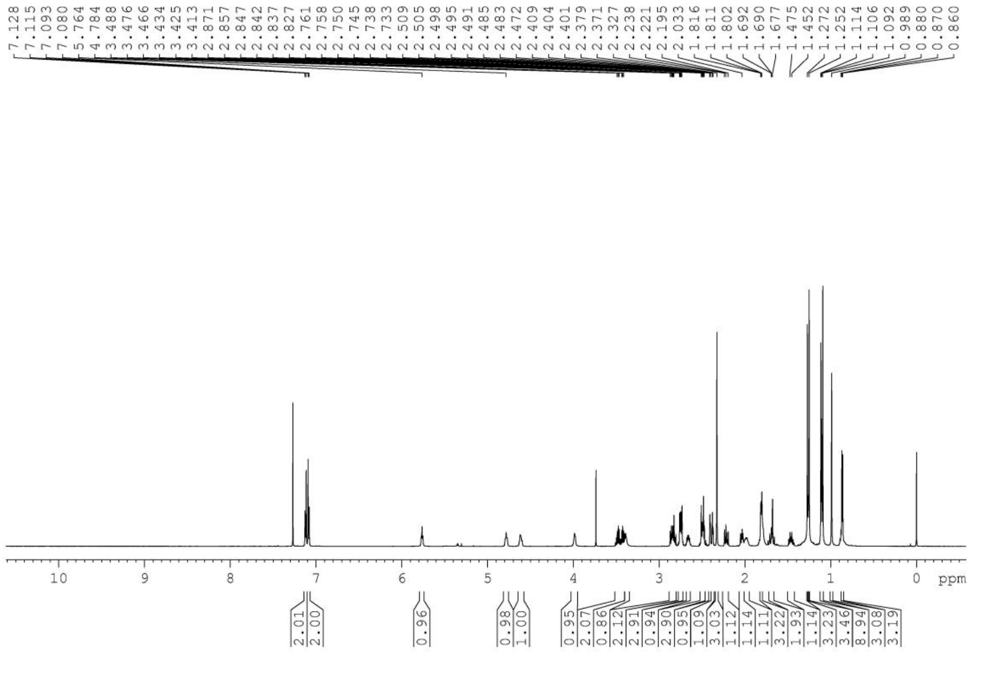

Supplementary Figure. S38 The ^{1}H NMR spectra of compound **A10**

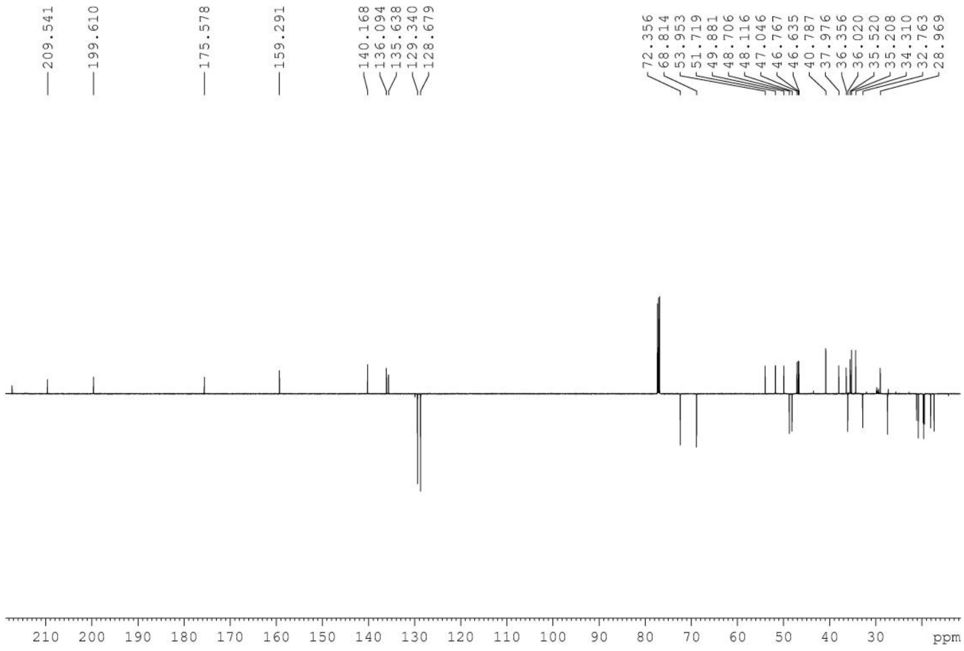

Supplementary Figure. S39 The ^{13}C NMR spectra of compound **A10**

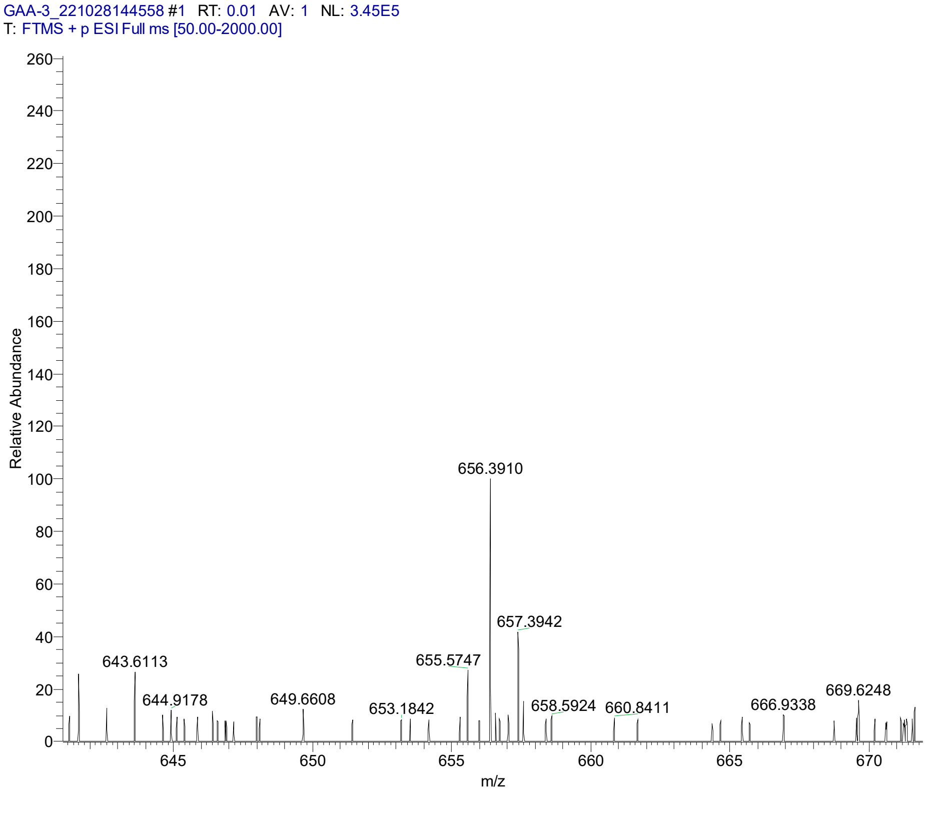

Supplementary Figure. S40 The HRMS spectra of compound **A10**

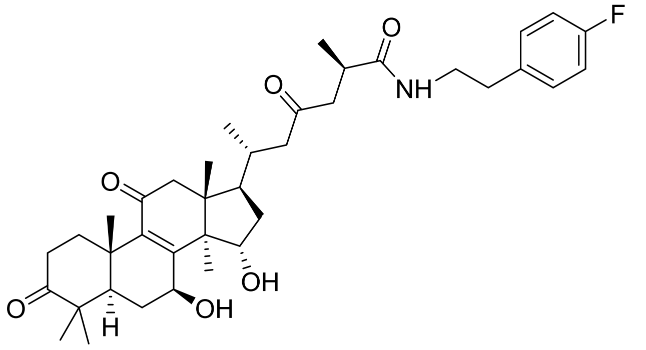

Supplementary Figure. S41 The chemical structure of compound **A11**. Chemical formula $C_{38}H_{52}ClNO_6$; molecular weight: 637.83

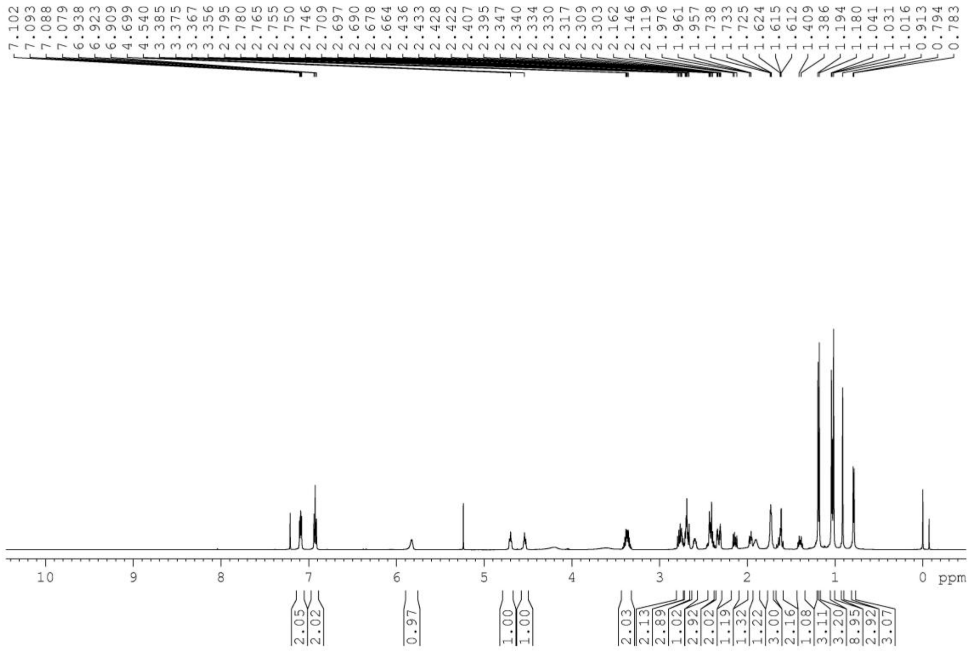

Supplementary Figure. S42 The ^{1}H NMR spectra of compound **A11**

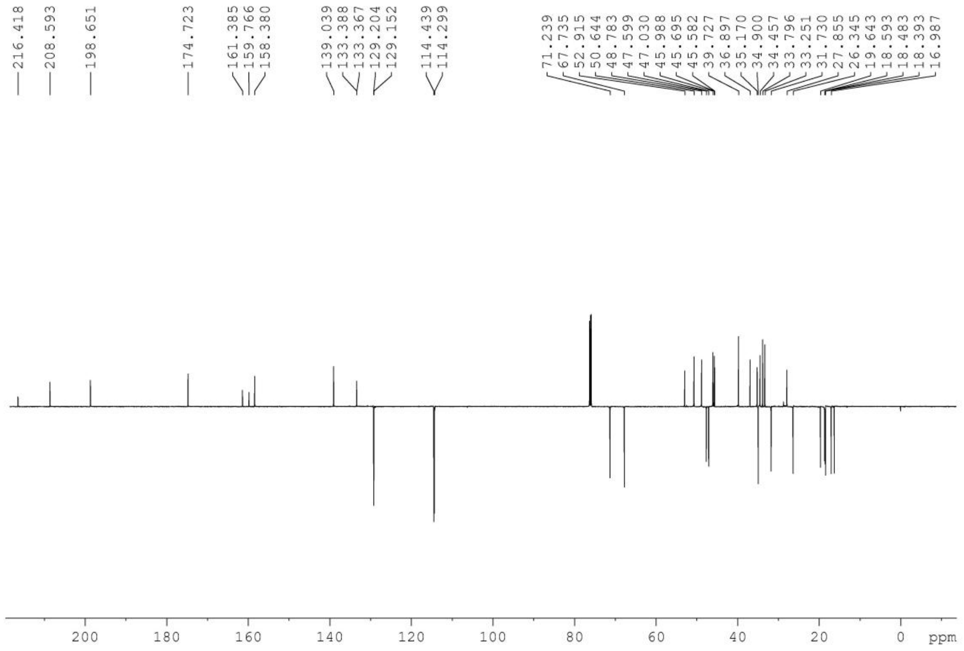

Supplementary Figure. S43 The ^{13}C NMR spectra of compound **A11**

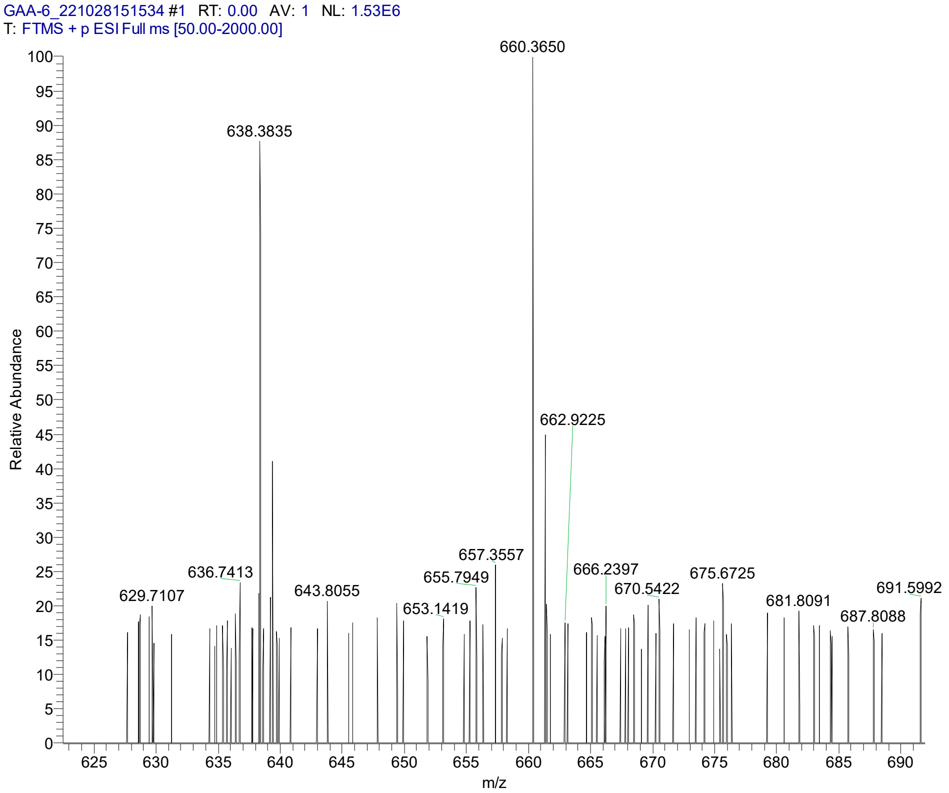

Supplementary Figure. S44 The HRMS spectra of compound **A11**

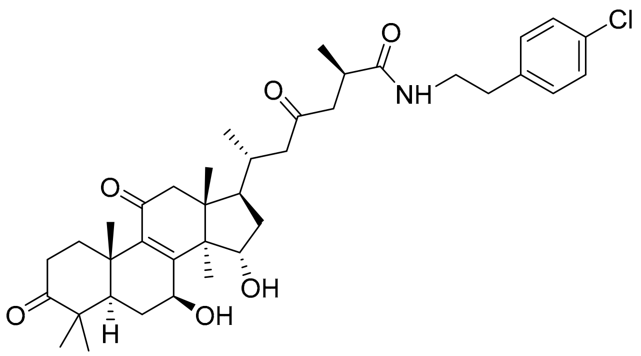

Supplementary Figure. S45 The chemical structure of compound **A12.** Chemical formula $C_{38}H_{52}ClNO_6$; molecular weight: 654.29

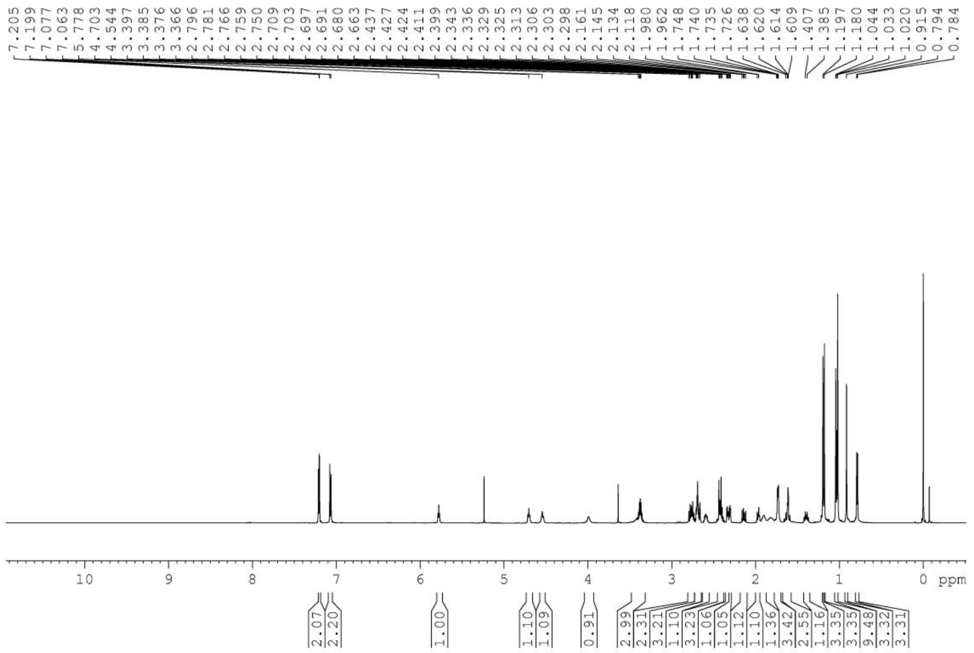

Supplementary Figure. S46 The ^{1}H NMR spectra of compound **A12**

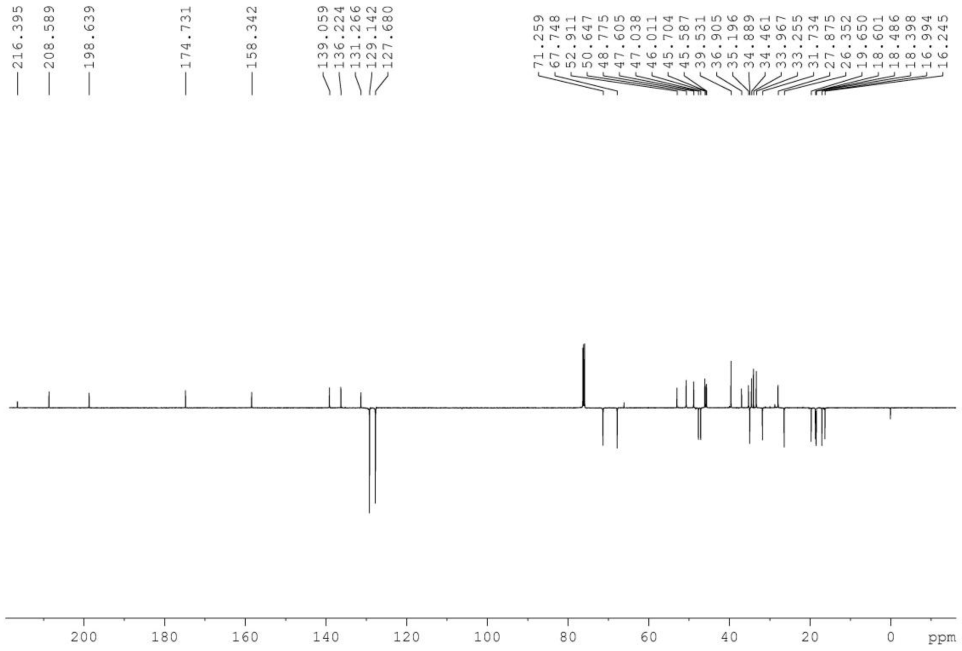

Supplementary Figure. S47 The ^{13}C NMR spectra of compound **A12**

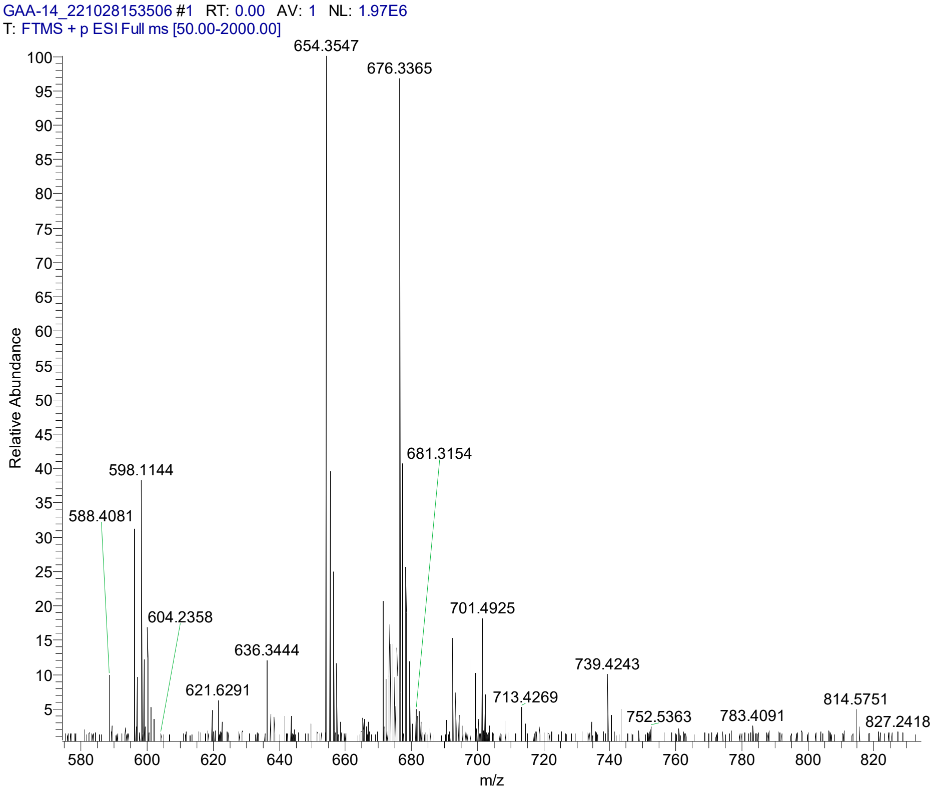

Supplementary Figure. S48 The HRMS spectra of compound **A12**

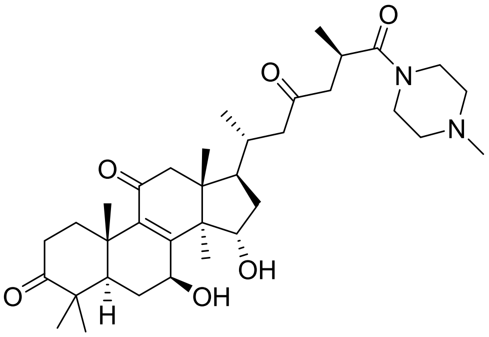

Supplementary Figure. S49 The chemical structure of compound **A13**. Chemical formula $C_{35}H_{54}N_2O_6$; molecular weight: 598.83

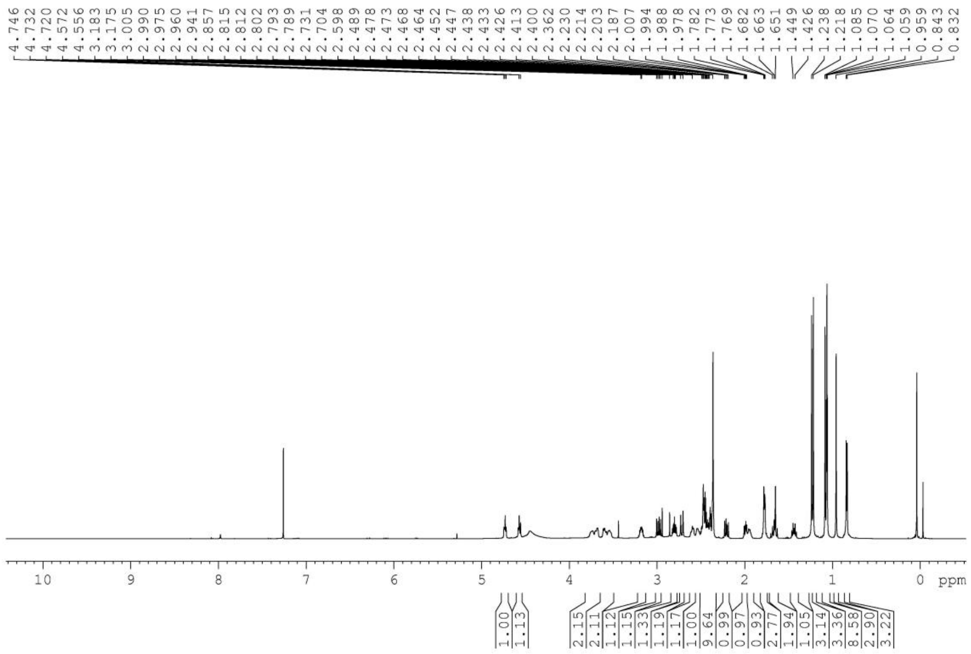

Supplementary Figure. S50 The ^{1}H NMR spectra of compound **A13**

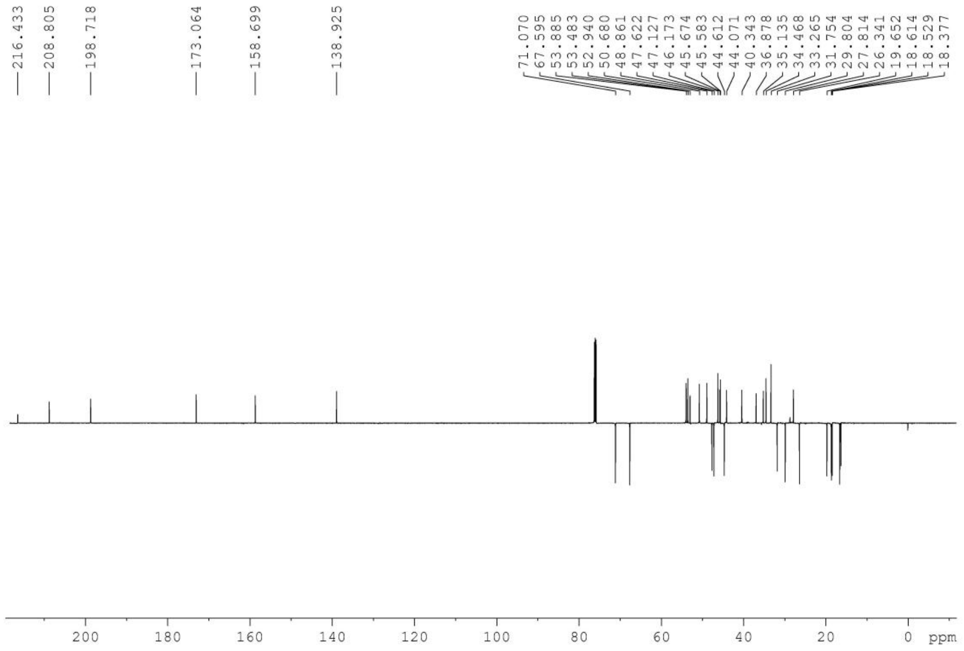

Supplementary Figure. S51 The ^{13}C NMR spectra of compound **A13**

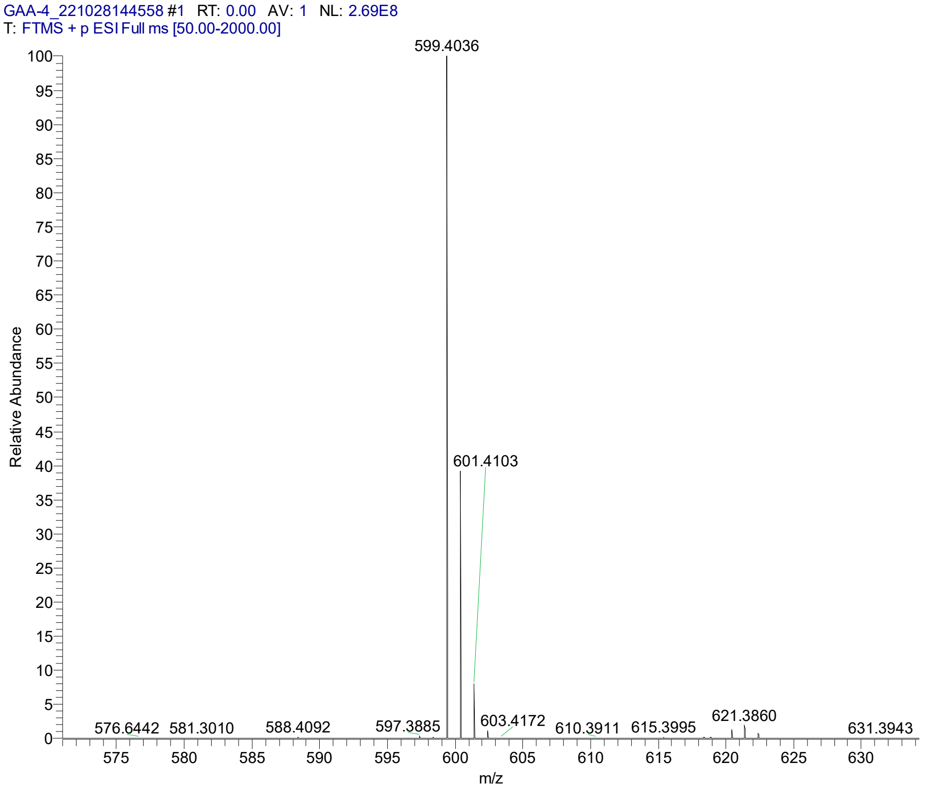

Supplementary Figure. S52 The HRMS spectra of compound **A13**

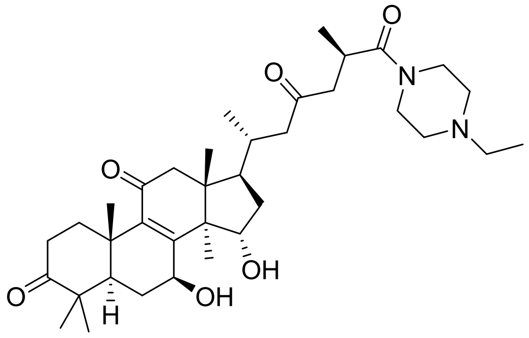

Supplementary Figure. S53 The chemical structure of compound **A14**. Chemical formula $C_{36}H_{56}N_2O_6$; molecular weight: 612.85

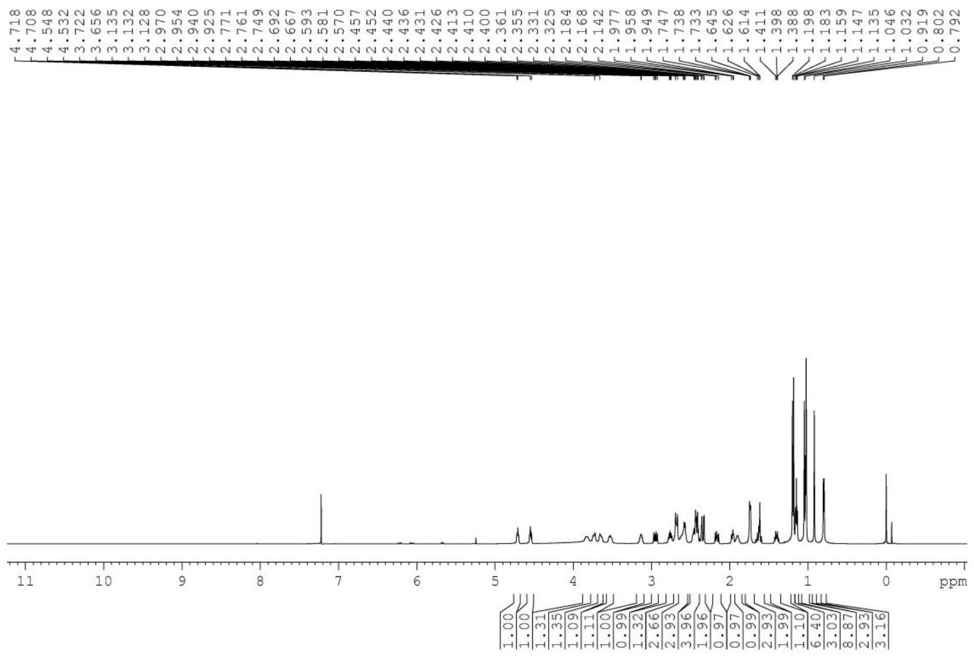

Supplementary Figure. S54 The ^{1}H NMR spectra of compound **A14**

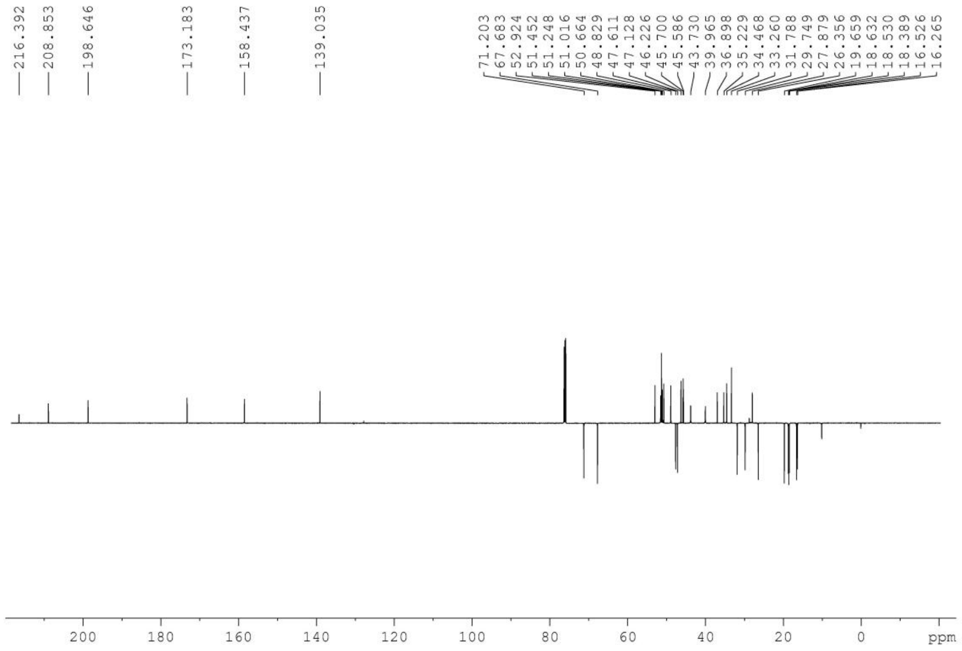

Supplementary Figure. S55 The ^{13}C NMR spectra of compound **A14**

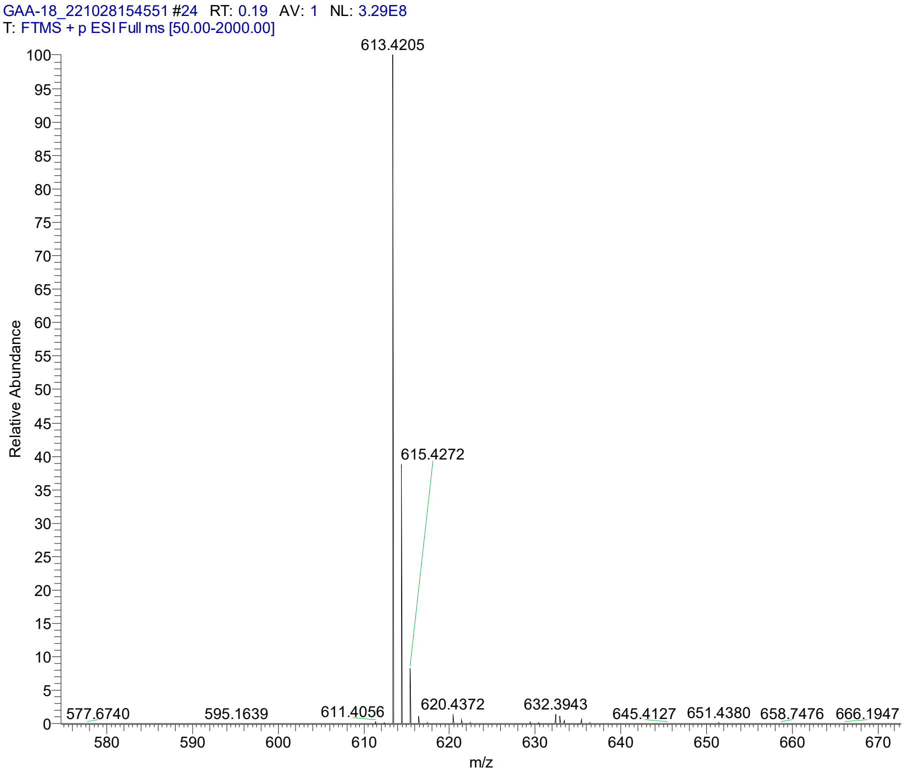

Supplementary Figure. S56 The HRMS spectra of compound **A14**

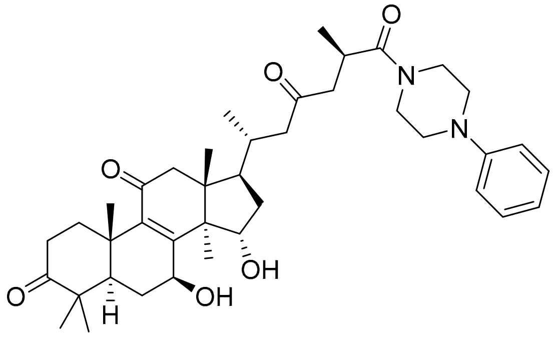

Supplementary Figure. S57 The chemical structure of compound **A15**. Chemical formula $C_{40}H_{56}N_2O$; molecular weight: 660.90

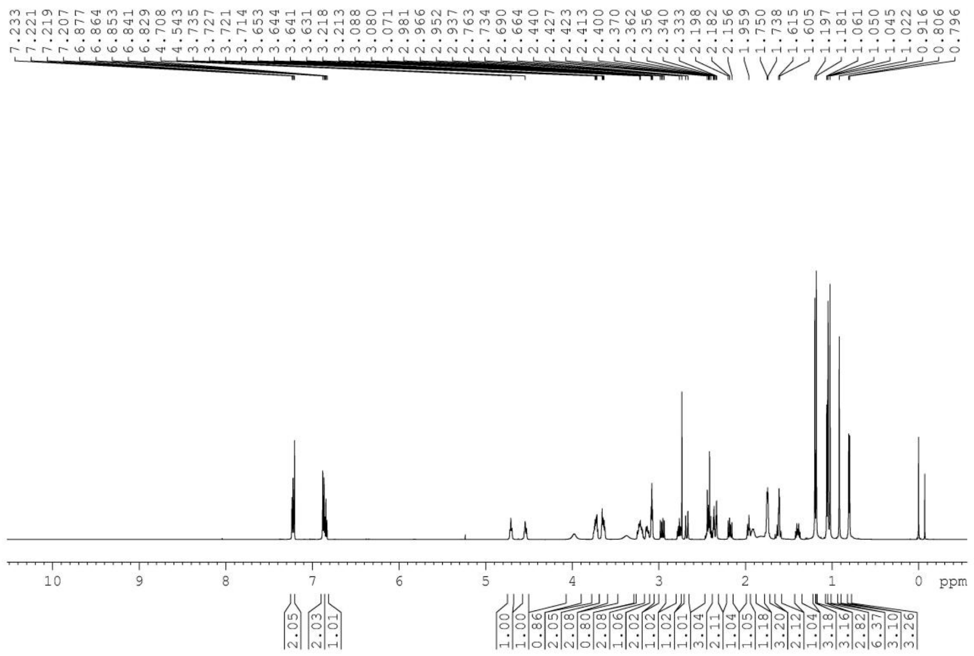

Supplementary Figure. S58 The ^{1}H NMR spectra of compound **A15**

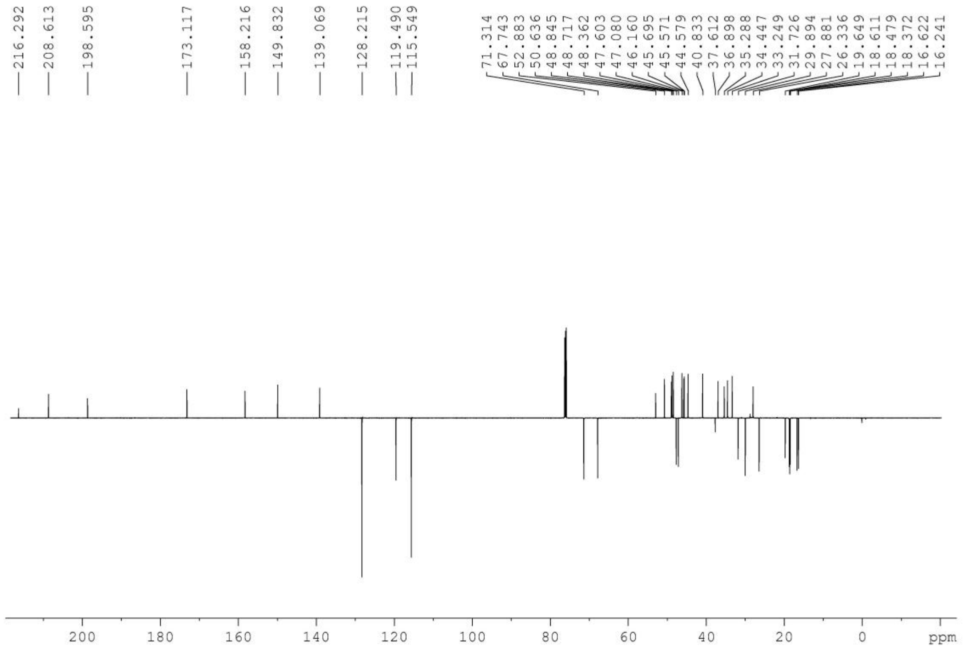

Supplementary Figure. S59 The ^{13}C NMR spectra of compound **A15**

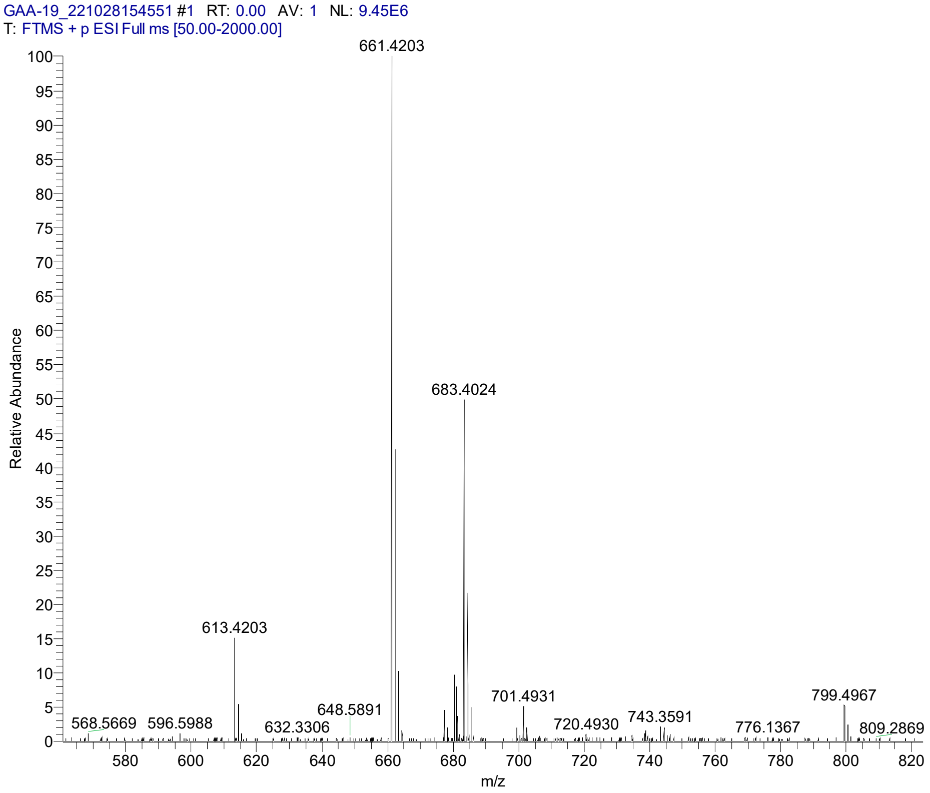

Supplementary Figure. S60 The HRMS spectra of compound **A15**
